# Critical roles of IKAROS and HDAC1 in regulation of heterochromatin and tumor suppression in T-cell acute lymphoblastic leukemia

**DOI:** 10.1038/s41375-025-02651-1

**Published:** 2025-06-24

**Authors:** Yali Ding, Bing He, Daniel Bogush, Joseph Schramm, Chingakham Singh, Katarina Dovat, Julia Randazzo, Diwakar Tukaramrao, Jeremy Hengst, Charyguly Annageldiyev, Avinash Kudva, Dhimant Desai, Arati Sharma, Vladimir S. Spiegelman, Suming Huang, Chi T. Viet, Glenn Dorsam, Giselle Saulnier Scholler, James Broach, Feng Yue, Sinisa Dovat

**Affiliations:** 1 Department of Pediatrics, Pennsylvania State University College of Medicine, Hershey, PA USA; 2 Department of Medicine, Pennsylvania State University College of Medicine, Hershey, PA USA; 3 Department of Pharmacology, Pennsylvania State University College of Medicine, Hershey, PA USA; 4 Department of Oral Maxillofacial Surgery, Loma Linda University School of Dentistry, Loma Linda, CA USA; 5 Department of Microbiological Sciences, North Dakota State University, Fargo, ND USA; 6 Department of Biochemistry and Molecular Biology, Pennsylvania State University College of Medicine, Hershey, PA USA; 7 Northwestern University Feinberg School of Medicine, Chicago, IL USA

**Keywords:** Cancer epigenetics, Acute lymphocytic leukaemia

## Abstract

The *IKZF1* gene encodes IKAROS – a DNA binding protein that acts as a tumor suppressor in T-cell acute lymphoblastic leukemia (T-ALL). IKAROS can act as a transcriptional repressor via recruitment of histone deacetylase 1 (HDAC1) and chromatin remodeling, however the mechanisms through which IKAROS exerts its tumor suppressor function via heterochromatin in T-ALL are largely unknown. We studied human and mouse T-ALL using a loss-of-function and *IKZF*1 re-expression approach, along with primary human T-ALL, and normal human and mouse thymocytes to establish the role of IKAROS and HDAC1 in global regulation of facultative heterochromatin and transcriptional repression in T-ALL. Results identified novel IKAROS and HDAC1 functions in T-ALL: Both IKAROS and HDAC1 are essential for EZH2 histone methyltransferase activity and formation of facultative heterochromatin; recruitment of HDAC1 by IKAROS is critical for establishment of H3K27me3 histone modification and repression of active enhancers; and IKAROS-HDAC1 complexes promote formation and expansion of H3K27me3 Large Organized Chromatin lysine (K) domains (LOCKs) and Broad Genic Repression Domains (BGRDs) in T-ALL. Our results establish the central role of IKAROS and HDAC1 in activation of EZH2, global regulation of the facultative heterochromatin landscape, and silencing of active enhancers that regulate oncogene expression.

## Introduction

IKAROS is a transcription factor which acts as a master regulator of hematopoiesis [1–3] and is essential for immune system development [4–9] and function [10, 11]. Loss of IKAROS function is associated with high-risk B-ALL [5, 12], T-ALL and early T-cell precursor (ETP) leukemia [13]. IKAROS can activate or repress transcription directly or via recruitment of chromatin remodeling complexes to the promoters of its target genes [14–26]. Several reports demonstrated that IKAROS can regulate gene expression in more complex ways – via global regulation of enhancer landscape [27], and by regulating global chromatin architecture [28].

Elucidating the mechanisms through which IKAROS regulates transcription of its target genes is essential for understanding its tumor suppressor function in leukemia. A majority of previous reports focused on the role of IKAROS in epigenetic regulation in normal lymphopoiesis and B-ALL, but studies regarding IKAROS function in the epigenomic regulation of transcription in T-ALL are limited [29–34]. IKAROS’ role in transcriptional repression of individual genes has been documented, however its function in global regulation of transcriptional repression in T-ALL has not been studied. Here we use an *IKZF*1 loss-of-function and re-expression approach in human and mouse T-ALL, along with primary human T-ALL, and normal human and mouse thymocytes to determine the roles of IKAROS and HDAC1 in global regulation of facultative heterochromatin and transcriptional repression in T-ALL. Results revealed novel roles of IKAROS and HDAC1 in regulation of EZH2 histone methyltransferase activity, enhancer silencing, and global regulation of formation and expansion of large heterochromatin domains. These data led to a new model of IKAROS function as a global regulator of heterochromatin landscape and gene expression in leukemia.

## Materials and methods

### Cell culture and viral transduction

JE131 cells (referred here as DN3) are *Ikzf1*-null early T-ALL cells that develop spontaneously in *Ikzf1* knock-out mice [35]. DN3 cells were transduced using an MSCV-based bicistronic retroviral vector that expresses IKAROS and GFP. Wild-type cells as well as retrovirally-transduced cells were collected at 1 and 2-day timepoints for experiments.

### RNA-seq

Total RNA was extracted using QIAGEN RNeasy Mini Kit (Qiagen, Hilden Germany). Libraries were generated and sequenced at the sequencing core facility at Pennsylvania State University College of Medicine (PSUCOM).

### ChIP-seq

ChIP-seq assays for IKAROS, EZH2, HDAC1 and histone modifications were performed using antibodies and methods described in Supplemental Data and as previously described [36, 37]. Samples were sequenced using the Illumina HiSeq 2500 at the sequencing core facility at PSUCOM. The rest of experimental procedures and bioinformatics analysis is described in Supplemental Data.

## Results

### IKAROS is essential for formation of facultative heterochromatin

**Fig. 1:**
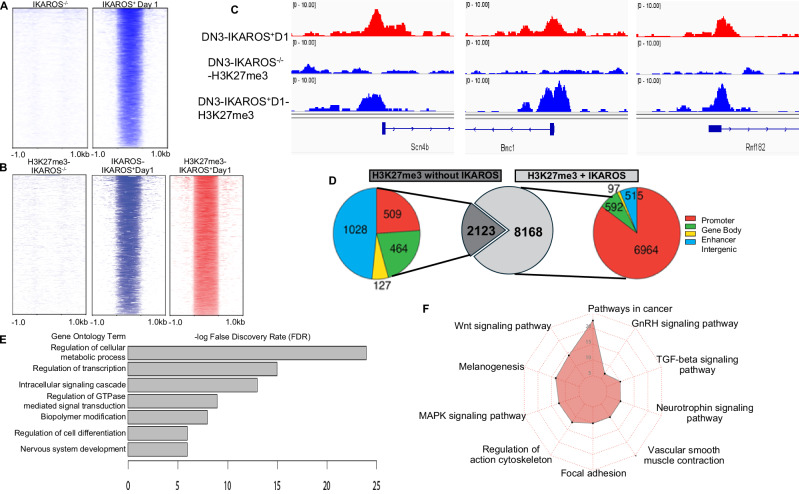
IKAROS binding induces de novo formation of facultative heterochromatin (H3K27me3). **A** Heatmaps of H3K27me3 ChIP-seq signals at day 1 after IKAROS expression vs. day 0. Signals are centered on H3K27me3 peaks at day 1. **B** Heatmaps of IKAROS and H3K27me3 ChIP-seq signals with IKAROS direct binding regions at day 0 vs. day 1. Signals are centered on IKAROS and H3K27me3 peaks at day 1. **C** Examples of de novo-formed H3K27me3 enrichment that are induced by IKAROS binding. **D** De novo H3K27me3 regions classified by function of the DNA element. **E**, **F** Gene ontology and pathway enrichment analysis of genes associated with de novo H3K27me3 regions.

The role of IKAROS in regulation of heterochromatin landscape in T-ALL was studied by comparing IKAROS loss of function and re-expression of *Ikzf1* into *Ikzf1*-null T-ALL cells as previously described (Fig. S1A) [27]. *Ikzf1*-null T-ALL cells show broad, weak H3K27me3 genome-wide occupancy with very few distinct peaks. Re-expression of IKAROS via retroviral transduction induces cellular growth arrest (Fig. S1B, C), along with abundant H3K27me3 enrichment throughout the genome, predominantly at gene promoters, within 1 day (Fig. 1A). A large majority of newly-enriched H3K27me3 overlaps IKAROS occupancy, suggesting that IKAROS DNA binding directly induces formation of facultative heterochromatin (Fig. 1B, C). Motif analysis shows enrichment of IKAROS’ core binding motif in the de novo-formed facultative heterochromatin (Fig. S2). Most IKAROS-induced de novo heterochromatin regions are located within promoters (Fig. 1C, D) of genes that regulate cellular metabolism, signal transduction, and oncogenic pathways (Fig. 1E, F).

Analysis of H3K27me3 enrichment following IKAROS-re-expression revealed a dual effect of IKAROS on the facultative heterochromatin landscape: 1) direct formation of facultative heterochromatin is associated with IKAROS occupancy and occurs mostly at the promoters of target genes and 2) establishment of global facultative heterochromatin landscape without IKAROS occupancy (indirect effect). Direct formation of facultative heterochromatin by IKAROS is predominant the first day following *Ikzf1* re-expression. In contrast, the global increase (over 4-fold) of H3K27me3 peaks in facultative heterochromatin is mostly independent of concomitant IKAROS DNA binding on day 2 following IKAROS re-expression with similar distribution among promoters, gene body and intergenic regions (Fig. S3A–C). Dynamic changes in the H3K27me3 signature during the 2 days following *Ikzf1* re-expression is consistent with establishment of the heterochromatin landscape that is found in normal thymocytes (Figs. S4A, B, S5). Data suggests that IKAROS is essential for formation of facultative heterochromatin and that IKAROS regulates H3K27me3 landscape both by direct DNA binding and indirectly, likely by influencing the activity of genes that regulate H3K27me3 formation. Genome-wide distribution of H3K27me3 following *Ikzf1* re-expression suggests IKAROS-driven heterochromatin re-programming to that of a physiological thymocyte.

### Interaction between EZH2 and IKAROS is required for formation of facultative heterochromatin

**Fig. 2:**
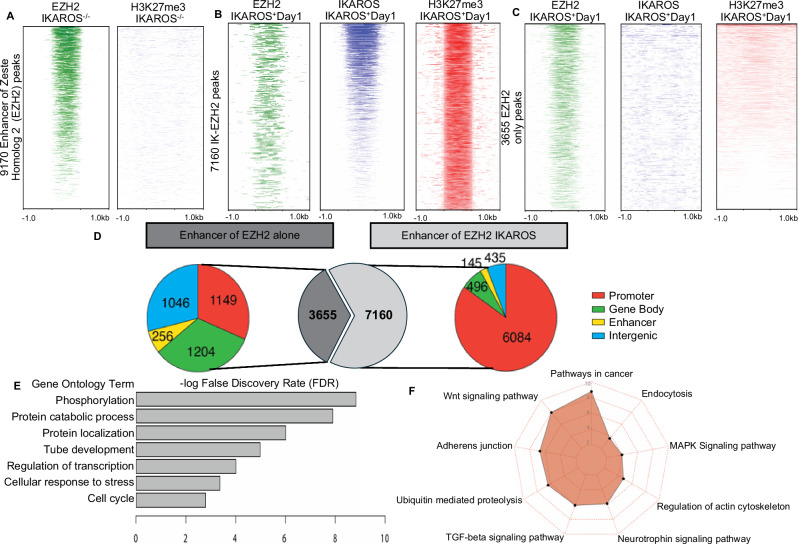
IKAROS recruits and activates EZH2. **A** Heatmaps of EZH2 and H3K27me3 ChIP-seq signals in IKAROS-null T-ALL. **B**, **C** Heatmaps of EZH2, IKAROS and H3K27me3 ChIP-seq signals in IKAROS-EZH2 occupied (**B**), and EZH2-only occupied (**C**) regions. **D** EZH2-only and EZH2-IKAROS occupied sites classified by function of the DNA element. **E**, **F** Gene ontology and pathway enrichment analysis of genes associated with EZH2-IKAROS complexes.

Enhancer of zeste homolog 2 (EZH2) is a histone methyltransferase that catalyzes the trimethylation of Histone 3 at lysine 27 resulting in H3K27me3 repressive chromatin [38, 39]. IKAROS can bind and recruit EZH2 to promoters of IKAROS target genes resulting in the formation of facultative heterochromatin [40]. We analyzed how the absence of *Ikzf1* and its re-expression affect function of EZH2. ChIP-seq showed abundant EZH2 occupancy in *Ikzf1-*null T-ALL, mostly at promoter regions, but without enrichment of H3K27me3 (Fig. 2A, S6A). Re-expression of *Ikzf1*, is associated with strong recruitment of EZH2 predominantly by IKAROS, and was associated with H3K27me3 enrichment (Fig. 2B-left heat maps, S6B). DNA-bound EZH2 without IKAROS association showed poor enrichment in H3K27me3 (Fig. 2C, S6C). Interestingly, many of the sites occupied solely by EZH2 in day 0 without H3K27me3 enrichment, were occupied by IKAROS-EZH2 complexes in day #1 and resulted in H3K27me3 enrichment (Fig. S6D). The relationship between EZH2 and IKAROS peaks in *Ikzf1*-null T-ALL and 1 day following *Ikzf1* re-expression is shown in Figure S7. Results in Fig. 2A–C suggest that EZH2 DNA occupancy without concomitant IKAROS DNA binding is not sufficient to induce H3K27me3 enrichment, and that EZH2 requires IKAROS binding for the formation of facultative heterochromatin. IKAROS-EZH2 complexes are mostly located at the promoters of their target genes (Fig. 2D), which regulate protein metabolism and signaling pathways in cancer (WNT, MAPK, etc.) (Fig. 2E, F).

Analysis of EZH2 enrichment in the subsequent day showed reduced occupancy of EZH2 and IKAROS-EZH2 complexes, especially at the promoters of target genes (Fig. S8). Overall, these data suggest that formation of IKAROS-EZH2 complex is essential for H3K27me3 enrichment and formation of facultative heterochromatin the first day after *Ikzf1* re-expression, and that EZH2 DNA binding in *Ikzf1*-null T-ALL is not associated with H3K27me3 enrichment.

### IKAROS recruits HDAC1 to the promoters of the genes that regulate malignant transformation

**Fig. 3:**
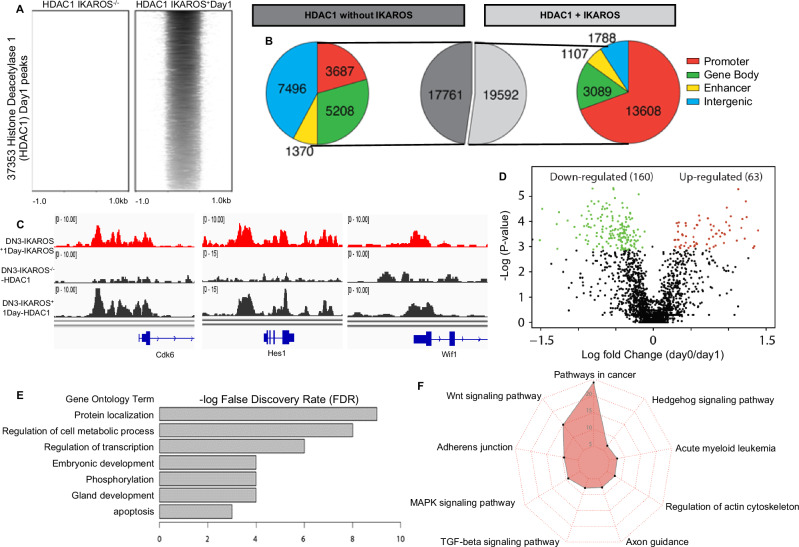
IKAROS is essential for HDAC1 recruitment to the gene promoters. **A** Heatmaps of HDAC1 ChIP-seq signals in IK-null T-ALL and day 1 following IKAROS re-expression. **B** HDAC1-only and HDAC1-IKAROS occupied sites classified by function of the DNA element. **C** Examples of IKAROS recruitment of HDAC1 to the promoters of IKAROS target genes. **D** Analysis of the differentially expressed genes directly regulated by IKAROS-HDAC1 complexes. **E**, **F** Gene ontology and pathway enrichment analysis of genes associated with IKAROS-HDAC1 complexes.

IKAROS can recruit HDAC1 to regulate expression of its target genes via chromatin remodeling [14, 15, 22, 41, 42]. We analyzed whether the lack of *Ikzf1* and its re-expression regulate HDAC1 genome-wide distribution and function. In *Ikzf1*-null T-ALL, HDAC1 DNA occupancy is poor and occurs mostly at intergenic regions (Fig. 3A and S9A-top panel). In day 1 following *Ikzf1* re-expression, there is a strongly increased HDAC1 DNA occupancy due to 1) recruitment of HDAC1 by IKAROS primarily to promoters (Fig. 3B, C and S9B, C); and 2) due to global genomic occupancy of HDAC1 independent of IKAROS binding (Fig. 3B, S9). IKAROS-HDAC1 complexes localize to promoters and are mostly associated with repression of their target genes that regulate protein metabolism and oncogenic signaling pathways (Fig. 3D–F), similarly to the pathways regulated by EZH2 and H3K27me3.

Analysis of HDAC1 occupancy in second day following *Ikzf1* re-expression showed highly increased HDAC1 genome-wide occupancy, with IKAROS-HDAC1 complexes present mostly at the promoters of IKAROS target genes. However, most HDAC1 global DNA enrichment was not associated with concomitant IKAROS binding, and is similarly distributed among promoters, gene body and intergenic regions (Fig. S9).

### HDAC1 has a critical role in formation of facultative heterochromatin

**Fig. 4:**
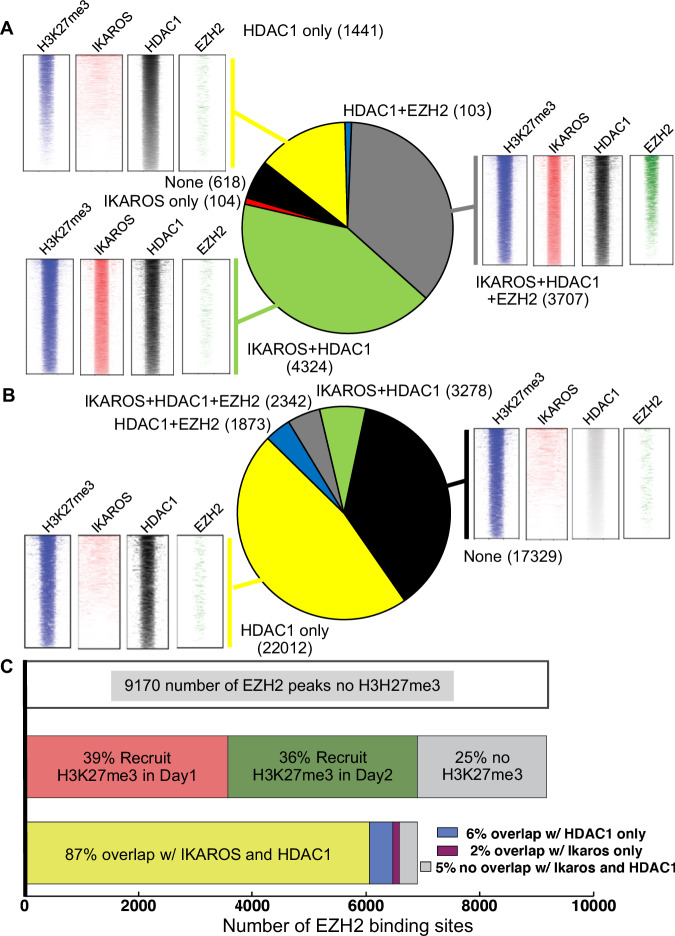
Critical roles of IKAROS and Histone Deacetylase 1(HDAC1) in the global de novo formation of H3K27me3. **A, B** IKAROS, HDAC1, and Enhancer of Zeste homologue 2(EZH2) occupancy associated with H3K27me3 formation Day 1 (**A**) and Day 2 (**B**) following I*kzf1* re-expression. **C** EZH2 target genes are regulated by the IKAROS and HDAC1-induced epigenetic switch. EZH2 DNA occupancy at target genes does not induce H3K27me3 in *Ikzf1*-null T-ALL (top). A large number (75%) of EZH2-target genes undergo epigenetic switch with de novo H3K27me3 formation following *Ikzf1*-re-expression (middle). Most of the EZH2 target genes that undergo epigenetic switch are regulated by IKAROS and HDAC1 binding (bottom).

We analyzed the relationship between IKAROS, EZH2 and HDAC1 with H3K27me3 enrichment and formation of facultative heterochromatin. Surprisingly, H3K27me3 enrichment has the strongest association with HDAC1 DNA occupancy, much stronger than occupancy by EZH2 and/or IKAROS (Fig. 4A, B) suggesting that HDAC1 has a prominent role in formation of H3K27me3, particularly at promoter regions (Fig. S10). Dynamic analysis of IKAROS, EZH2, and HDAC1 occupancy with H3K27me3 showed that initial formation of H3K27me3 (day 1) is associated either with IKAROS/HDAC1/EZH2 concomitant DNA binding, or with IKAROS/HDAC1 DNA binding without EZH2 occupancy (Fig. 4A–D). However, further H3K27me3 enrichment (day 2) is either associated with HDAC1 occupancy independent of IKAROS and/or EZH2 enrichment, or independent of IKAROS/HDAC1/EZH2 DNA binding altogether (Fig. 4B). HDAC1 inhibition following *IKZF1* re-expression severely reduces number of H3K27me3 peaks (Fig. S10D–F). These results strongly suggest that the recruitment of HDAC1 by IKAROS is essential for induction of H3K27me3 and that HDAC1 is critical for the maintenance of H3K27me3.

The role of HDAC1 recruitment by IKAROS, as well as global genomic occupancy of HDAC1 is highly prominent following *Ikzf1* re-expression and proliferation arrest of *Ikzf1*-null T-ALL cells. Since EZH2 DNA binding did not induce formation of H3K27me3 in *Ikzf1*-null cells, we analyzed whether IKAROS/HDAC1 complexes induce H3K27me3 enrichment at sites that were occupied by EZH2 in IKAROS-null T-ALL. Results showed that IKAROS re-expression induced H3K27me3 enrichment at 75% of the sites occupied by EZH2 in *Ikzf1*-null cells (Fig. 4C-middle). Almost all H3K27me3-enriched sites were occupied by both IKAROS and HDAC1 (Fig. 4C-bottom) and most were located at the promoters of genes that regulate oncogenic signaling pathways (Fig. S11A–D). HDAC1 directly interacts with EZH2 only when IKAROS is expressed (Fig. S11E). These results suggest that recruitment of HDAC1 by IKAROS is essential for EZH2 tumor suppressor function, including formation of facultative heterochromatin at promoters of genes that positively regulate oncogenic pathways.

### IKAROS and HDAC1 modulate activity of enhancers

**Fig. 5:**
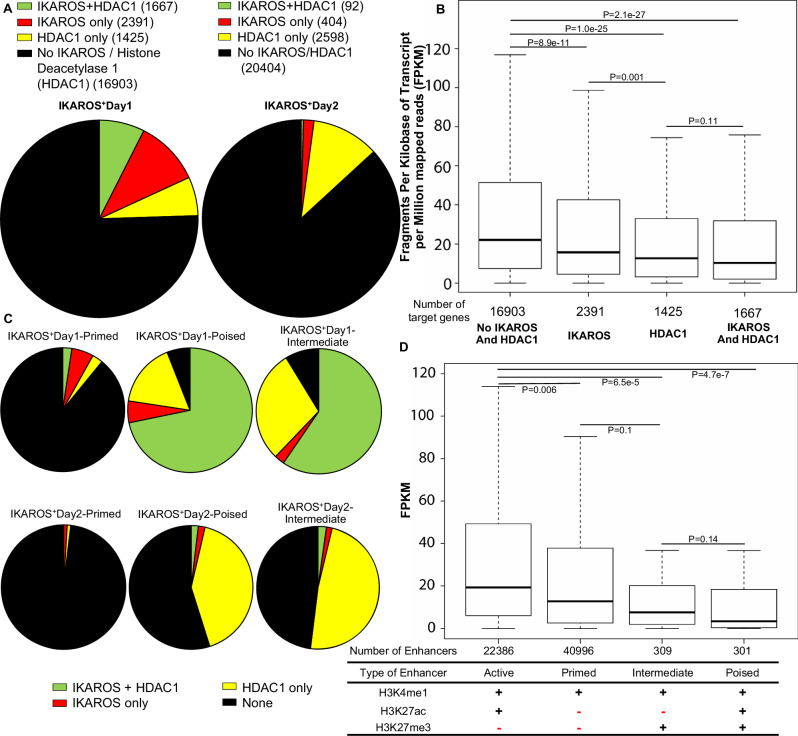
IKAROS and HDAC1 are repressors of active enhancers. **A** A large number of active enhancers are occupied by IKAROS and HDAC1 after IKAROS re-expression. Pie charts showing the portions of active enhancers occupied by IKAROS, HDAC1, or both IKAROS and HDAC1 (**B**) Boxplot shows expression of genes regulated by the active enhancers not bound by IKAROS and/or HDAC1 (left) and occupied by IKAROS and/or HDAC1. **C** IKAROS and HDAC1 regulate formation of poised and intermediate enhancers. Pie charts showing the portions of primed, poised, and silenced enhancers occupied by IKAROS, HDAC1, or both IKAROS and HDAC1 in day 1 and 2 following *Ikzf1* re-expression. IKAROS-HDAC1 co-occupancy is highly associated with formation of poised and intermediate enhancers in day 1, while HDAC1-only occupancy is dominant in those enhancers in day 2. **D** Boxplot shows expression of genes regulated by the active, primed, intermediate, and poised enhancers.

Next, we analyzed how recruitment of HDAC1 by IKAROS regulates enhancer activity. A large set of enhancers are occupied by either IKAROS or HDAC1 following *Ikzf1* re-expression into *Ikzf1*-null cells (Fig. 5A). We compared gene transcription regulated by active enhancers (H3K4me1 + /H3K27ac + ) without IKAROS or HDAC1 occupancy (IKAROS-/HDAC1-) to transcription regulated by enhancers with enrichment for IKAROS (IKAROS + ), HDAC1 (HDAC1 + ) or both IKAROS and HDAC1 (IKAROS + /HDAC1 + ). IKAROS occupancy at active enhancers has a repressive effect on transcription of genes targeted by those enhancers (Fig. 5B). HDAC1 enrichment has an even more pronounced repressive effect on enhancer activity, similar to concomitant IKAROS and HDAC1 enrichment (Fig. 5B). Modulation of enhancer function by IKAROS and HDAC1, represses the transcription of genes that regulate cancer pathways (Figs. S12–14).

Non-active enhancers can be classified as primed (enriched in H3K4me1+ only), intermediate (H3K4me1 + /H3K27ac + /H3K27me3 + ) and poised (H3K4me1 + /H3K27me3 + ) [43, 44]. We analyzed whether IKAROS and HDAC1 can regulate formation of these enhancer types. Results show that a majority of poised and intermediate enhancers in day 1 following IKAROS re-expression are associated with both IKAROS/HDAC1 occupancy, while HDAC1 occupancy has a dominant role in formation of poised and intermediate enhancers during the second day following IKAROS re-expression into IKAROS-null T-ALL (Fig. 5C). Genes regulated by poised and intermediate enhancers have significantly reduced transcription compared to the genes regulated by active or primed enhancers (Fig. 5D). These data show that IKAROS and HDAC1 occupancy can silence enhancer activity in two ways: 1) by repressing activity of active enhancers; and 2) by inducing formation of poised and/or silenced enhancers. To study the functional significance of IKAROS and HDAC1 regulation of enhancer activity in T-ALL, we analyzed gene regulation by active enhancers in IKAROS-null T-ALL that become occupied by IKAROS and/or HDAC1 following IKAROS transduction (Fig. S15). Pathway enrichment analysis shows that these enhancers regulate genes involving pathways in cancers and cell proliferation (Fig. S15A). IKAROS re-expression and IKAROS/HDAC1 binding to those enhancers is associated with repression of many genes that promote stemness and drug resistance, e.g., adrenomedullin (Fig. S15B). These data suggest that IKAROS exerts its tumor suppressor function in T-ALL by recruiting HDAC1 and silencing activity of enhancers that regulate transcription of various oncogenes. These results identify the enhancers that are directly regulated by IKAROS in T-ALL and their corresponding pathways.

### IKAROS regulates formation and expansion of large organized chromatin lysine (K) domains (LOCKs)

**Fig. 6:**
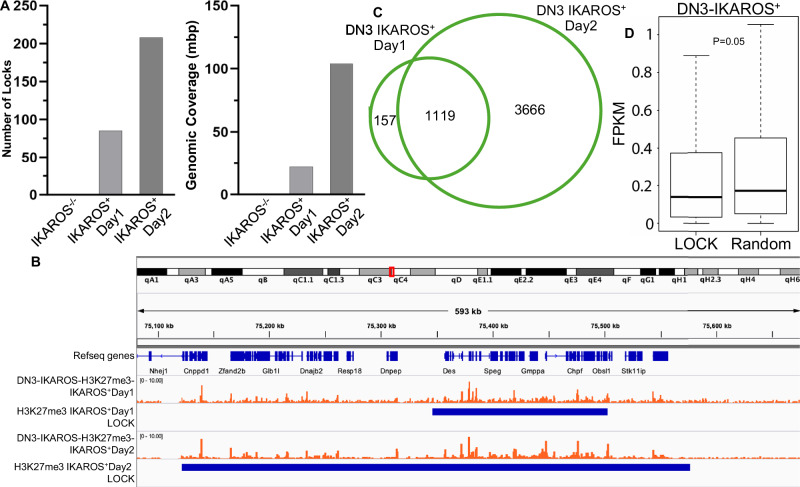
IKAROS regulates formation and expansion of H3K27m3 Long organized Chromatin Lysine(K) domains(LOCKs). **A** Number (left) and genomic coverage (right) of H3K27me3 LOCKs in day 1 and 2 following *Ikzf1* re-expression. **B** Example of the genomic expansion of the H3K27me3 LOCKs from day 1 to 2 after *Ikzf1* re-expression. **C** Number of genes regulated by the H3K27me3 LOCKs in day 1 and 2 following *Ikzf1* re-expression. **D** Gene expression of the genes regulated by LOCKs vs. random genes.

Global epigenetic landscape contains clusters of nucleosomes with a large number of post-transcriptionally modified lysine residues Large Organized Chromatin lysine(K) domains (LOCKs). LOCKS are associated with repression if they consist predominantly of heterochromatin marks (H3K27me3 or H3K9me3) [45]. We analyzed whether IKAROS can regulate formation and distribution of LOCKs. *Ikzf1* expression strongly induces de novo formation of H3K27me3 LOCKs, which becomes even more prominent 2 days following *Ikzf1* re-expression (Fig. 6A). Re-expression of *Ikzf1* increases the number of H3K27me3 LOCKs but has a greater effect by increasing the genomic coverage of H3K27me3 LOCKs (Fig. 6A). This is mostly due to strong day 2 expansion of de novo formed H3K27me3 LOCKs in day 1 (Fig. 6B). Expansion of the H3K27me3 LOCKs over 2 days following *Ikzf1* re-expression is associated with a large number of genes being regulated by LOCKs. (Fig. 6C). There is a dynamic shift in the function of the genes regulated by H3K27me3 LOCKs: in day #1 following IKAROS re-expression, H3K27me3 LOCKs regulate mostly pathways in cancer and cellular proliferation, while in day #2, H3K27me3 LOCKs mostly regulate genes involved in immune response (Fig. S16A, B). Expression of genes regulated by H3K27me3 LOCKs reduced compared to the expression of the random genes (Fig. 6D). These data demonstrate that IKAROS has an important role in organization of higher heterochromatin structures and gene expression via regulation of formation and expansion of H3K27me3 LOCKs.

### Regulation of heterochromatin landscape by IKAROS in T-ALL is evolutionarily conserved

**Fig. 7:**
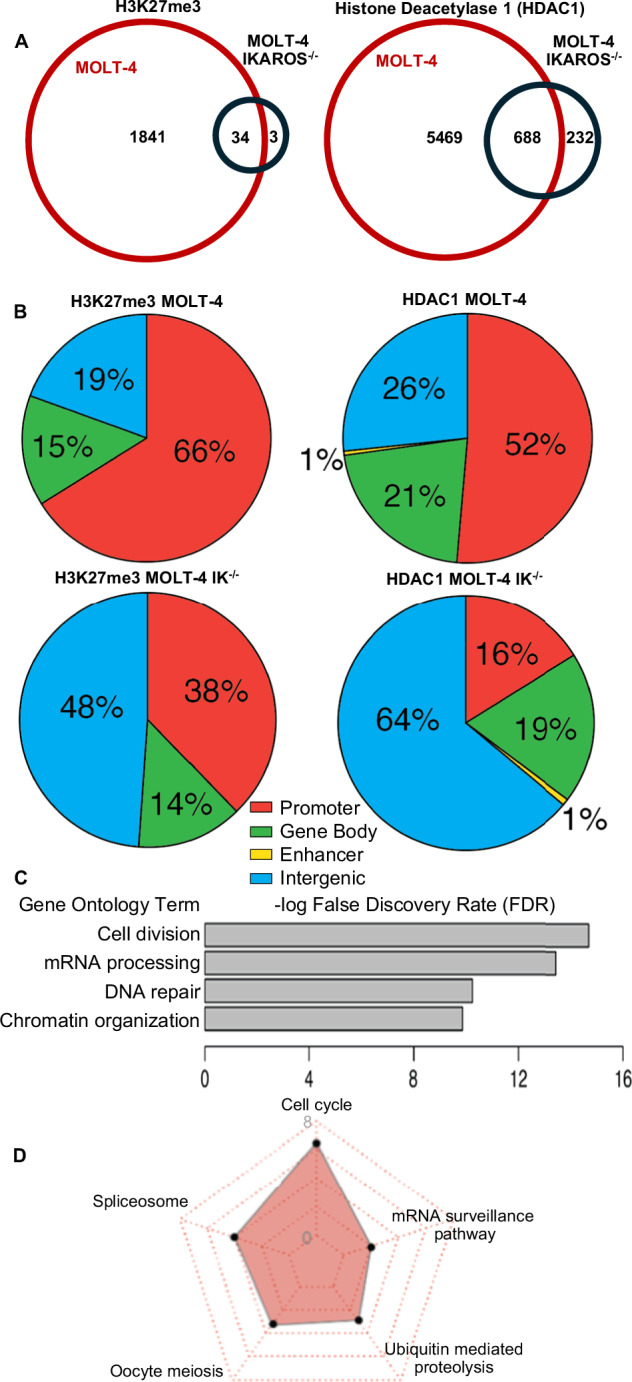
IKAROS regulates genomic occupancy and distribution of EZH2 and HDAC1 and H3K27me3 formation in human T-ALL. **A** Number of H3K27me3 (left) and HDAC1 (right) peaks in wildtype MOLT-4 and in *IKZF1*-knockout (MOLT-IK-KO) cells. **B** H3K27me3 and HDAC1 occupied sites, in wildtype MOLT-4 and in *IKZF1*-knockout (MOLT-IK-KO) cells, classified by function of the DNA element. **C**, **D** Gene ontology and pathway enrichment analysis of genes associated with HDAC1 occupancy in wildtype MOLT-4 cells.

IKAROS functions as a tumor suppressor in human T-ALL [13]. We tested whether IKAROS regulates heterochromatin landscape similarly in mouse and human T-ALL. The *IKZF*1 gene was deleted in human T-ALL cells MOLT-4 using the CRISPR-Cas system to create MOLT-4 *IKZF*1-null cells. We analyzed IKAROS’ role in heterochromatin regulation in human T-ALL by comparing the heterochromatin landscape of MOLT-4-*IKZF*1-null and MOLT-4 wild type cells. *IKZF*1 deletion in MOLT-4 cells resulted in reduced global H3K27me3 and HDAC1 occupancy (Fig. 7A). This was associated with the global genomic redistribution of H3K27me3 and HDAC1 due to their reduced occupancy at promoter regions (Fig. 7B, Fig. S17). *IKZF1* knockout reduced the H3K27me3 and HDAC1 occupancy at promoters of genes that regulate cell cycle progression, cellular division, and nucleotide metabolism (Fig. 7C, D). Gene expression analysis showed reduced expression of genes regulated by active enhancers with IKAROS and HDAC1 occupancy compared to gene expression regulated by active enhancers not occupied by IKAROS and HDAC1 in human T-ALL (Fig. S18). *IKZF1* deletion in MOLT-4 cells reduced both the number and the genomic coverage of H3K27me3 LOCKS (Fig. S19). Lack of IKAROS reduces the number of genes regulated by LOCKs, including genes involved in several oncogenic pathways (Fig. S20). We analyzed the effect of *IKZF1* deletion on formation of the Broad Genic Repression Domains (BGRD) in MOLT-4 cells. BGRDs are domains enriched in H3K27me3 (Fig. S21A) that are also enriched in oncogenes [46]. Expression of genes located within BGRDs is strongly reduced compared to random genes (Fig. S21B). *IKZF1* deletion reduces the number of BGRDs and its genomic coverage (Fig. S21C, D). We analyzed the H3K27me3 ChIP-seq data from mouse *Ikzf1*-null T-ALL on day 1 and 2 following *Ikzf1* re-expression and human primary T-ALL, and compared them with the published H3K27me3 genomic occupancy in normal mouse and human thymocytes. Comparative analysis of genome wide enrichment of H3K27me3 in normal and malignant human and mouse cells showed a remarkable conservation of H3K27me3 landscape in human and mouse thymocytes, loss of H3K27me3 in T-ALL, and an important role of IKAROS in maintaining H3K27me3 landscape (Fig. S22). These data suggest that IKAROS regulates global heterochromatin landscape in a similar way for both mouse and human T-ALL, validating the mouse T-ALL model used for this study and providing insight into the tumor suppressor role of IKAROS in human T-ALL.

## Discussion

**Fig. 8:**
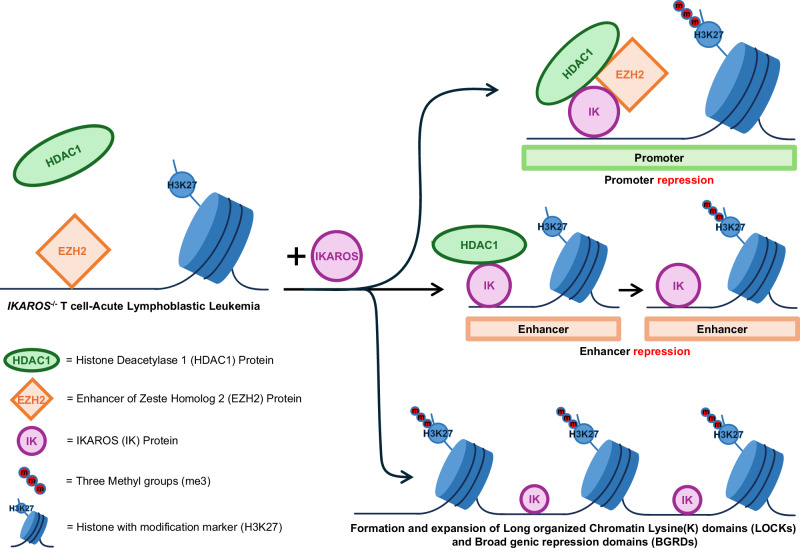
Novel IKAROS functions in the regulation of heterochromatin formation. IKAROS expression regulates: de novo formation of H3K27me3 by HDAC1 recruitment and activation of EZH2; repression of active enhancers; and formation and expansion of LOCKs and BGRDs.

IKAROS is a tumor suppressor in T-ALL. *Ikzf1* loss-of-function and re-expression experiments in human and mouse T-ALL, coupled with extensive epitranscriptomic analysis of primary T-ALL and thymocytes, identified novel mechanisms that regulate tumor suppression in T-ALL (Fig. 8):

- *Critical role of IKAROS-HDAC1 complexes in regulation of global facultative heterochromatin landscape*. Loss of the H3K27me3-associated heterochromatin has been associated with the development of T-ALL and other types of leukemia [13, 47–52]. Inactivating mutations of histone methyltransferase *EZH2* is considered the principal mechanism responsible for the loss of H3K27me3 in T-ALL [53–56]. Presented data revealed that DNA binding of EZH2 in the absence of IKAROS and/or HDAC1 co-localization is rarely associated with H3K27me3 in both *Ikzf1*-null and *Ikzf1*-wildtype mouse and human T-ALL. Lack of IKAROS is associated with severe depletion of DNA-bound HDAC1, and loss of EZH2-associated H3K27me3. Thus, HDAC1 recruitment by IKAROS is important for both EZH2 activity and H3K27me3 formation.
- *Central role of HDAC1 in EZH2 activation, H3K27me3 formation and maintenance*. Results showed that the recruitment of HDAC1 by IKAROS is essential for EZH2 function and de novo H3K27me3 formation. Since IKAROS recruits both HDAC1 and EZH2, and HDAC1 can interact with EZH2 [57], it is possible that HDAC1 directly activates EZH2 while in complex with IKAROS. HDAC1 DNA occupancy does not result in loss of H3K27ac, but rather in de novo formation and genome-wide expansion of H3K27me3.
  The data presented suggest that Ikaros exerts its tumor suppressor function in T-ALL by recruitment of HDAC1. This does not contradict the oncogenic role of HDAC1 in T-ALL. HDAC1 inhibitors showed strong efficacy against PTCL and ATLL. Presented results suggest that oncogenic role of HDAC1 in T-cell malignancies might predominantly involve the direct HDAC1 binding and deacetylation of non-histone HDAC1 target proteins that are critical for cellular proliferation (p53, pRB), DNA-repair proteins (Ku70, BRCA1, and RAD51), and chaperone proteins, instead of transcriptional regulation.
- *IKAROS/HDAC1 complexes can repress active enhancers and promote formation of intermediary and poised enhancers*. IKAROS can induce de novo formation of enhancers and activation of primed enhancers [27]. Presented data showed that IKAROS binding is associated with silencing of enhancers’ activity. This can occur via recruitment of HDAC1 without H3K27me3 formation (Fig. 5B), or via formation of intermediate or poised enhancers (Fig. 5D). These results, together with previously reported data, reveal a complex regulatory role of IKAROS in enhancer activity: IKAROS DNA binding can result in de novo formation and/or activation of enhancers, but also in repression of enhancers’ activity. IKAROS-silenced enhancers regulate oncogenic pathways in IKAROS-null T-ALL (Fig. S15), which suggests that silencing enhancers that regulate expression of oncogenes is one of the mechanisms through which IKAROS exerts a tumor suppressor function in T-ALL.
- *Regulation of formation and expansion of LOCKs and BGRDs*. Lack of IKAROS is associated with severe depletion of H3K27me3 LOCKs and BGRDs. These recently identified large heterochromatin domains (BGRDs) strongly repress large sets of genes, including oncogenes, and have a prominent role in global epigenomic regulation of gene expression [45, 46]. IKAROS-driven HDAC1 recruitment and H3K27me3 expansion is a likely mechanism through which IKAROS promotes formation of LOCKs and BGRDs. Results suggest that IKAROS-HDAC1 complexes could function as a tipping point at the intersection between euchromatin and heterochromatin in T-ALL. IKAROS’ prominent role in both formation of super-enhancers and large heterochromatin domains, suggest that IKAROS regulates higher chromatin organization.

In conclusion, our results identify novel functions of IKAROS and HDAC1 in regulation of heterochromatin propagation and enhancer activity. The presented data demonstrate that IKAROS-HDAC1 complexes regulate the balance between euchromatin and heterochromatin in T-ALL. Results reveal the novel mechanisms that regulate interplay between active and repressive chromatin, global regulation of gene expression, and tumor suppression in leukemia.

## Supplementary information

Supplemental Materials

## Data Availability

Sequencing data are deposited in NCBI GEO (GSE261180 and GSE261181).
