# Supplementary material for: Critical roles of IKAROS and HDAC1 in regulation of heterochromatin and tumor suppression in T-cell acute lymphoblastic leukemia
Source: Leukemia. 2025 Jun 24;39(8):2010–20. doi: 10.1038/s41375-025-02651-1 (PMC12310526; doi:10.1038/s41375-025-02651-1)
Supplement: Supplementary file 1 — Supplemental Materials [file 41375_2025_2651_MOESM1_ESM.pdf]

Fig S1 Experimental Design

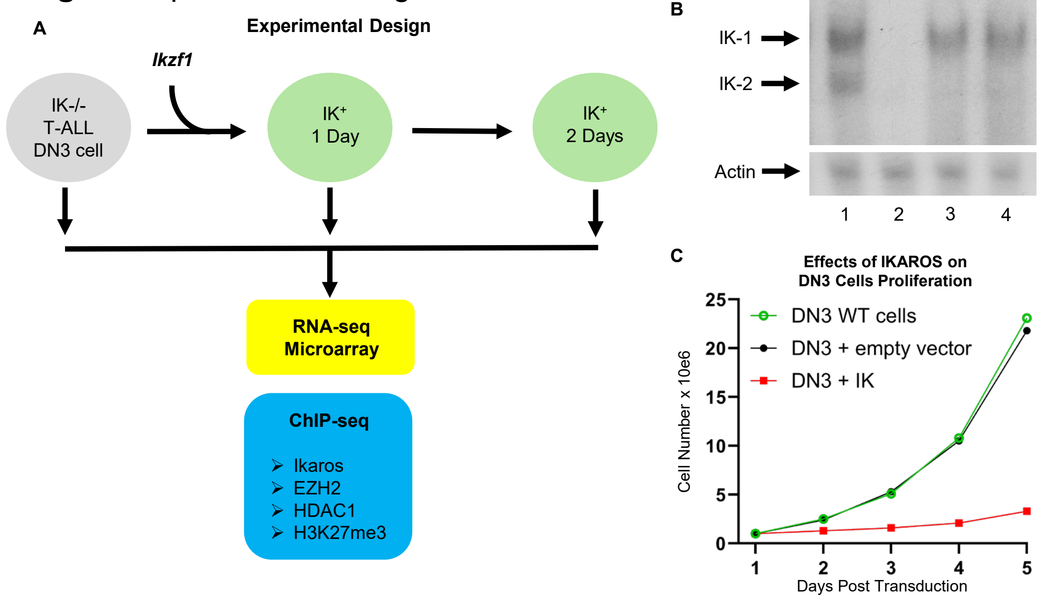

Supplemental Figure 1. (A) Experimental design. Ikaros (IK)-null murine T-ALL cells were transduced by *Ikzf1*-containing retrovirus to express IKAROS. Cells were cultured for 2 Days following retroviral transduction. Cells were harvested prior to transduction (Day 0) and at daily time points over the 2 Day period (Day 1 and Day 2) for the indicated analyses. **(B) Expression of IKAROS in DN3 cells.** Western blot analysis of IKAROS expression in primary mouse thymocytes (lane 1), DN3 cells transduced with empty retrovirus (lane 2) and DN3 cells following re-introduction of IK-1-HA-tagged in day 1 and 2 (Lanes 3-4 respectively). Western blot analysis shows that the amount of IKAROS protein in DN3 cells following retroviral re-introduction of Ik-1-HA tagged (lanes 3-4) is comparable to the amount of IK-1 in thymocytes (lane 1). The 2 largest IKAROS isoforms and transduced IK-1-HA tagged are indicated by arrows. Top panels show actin as a loading control **(C) Effect of *Ikzf1* re-introduction on proliferation of DN3 cells.** Ikaros (IK) -/- murine T-ALL cells were left untreated (green); transduced with MSCV-GFP empty vector (black) or transduced with MSCV-GFP retrovirus that express *Ikzf1* (red). Transduced cells expressing *Ikzf1* or empty retrovirus were isolated and cultured. Counts for viable cells were obtained every 24 hours by hemocytometer count with trypan blue staining. Graphed are the cell numbers at Days #1-#5.

Fig S2

Motif	P-value	Best Match TF
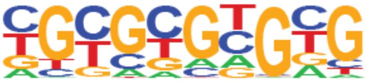	1e-20	EGR1
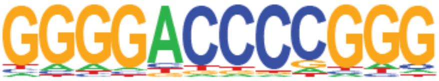	1e-14	ZFP165, *Ikzf1*/IKAROS
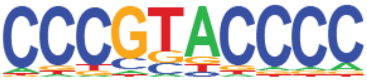	1e-13	ZFP281, SP1

Supplemental Figure 2. Motif enrichment analysis for de novo formed facultative heterochromatin (H3K27me3) peaks following *Ikzf1* introduction at Day 1 MEME-ChIP was used to extract the enriched known transcription factor binding motifs from a large set of DNA sequences identified by ChIP-seq by searching against the JASPAR CORE database. Motif logo, significance of the motif, and names of transcription factors that bind the motifs are shown.

Fig S3. Dynamic changes of H3K27me3

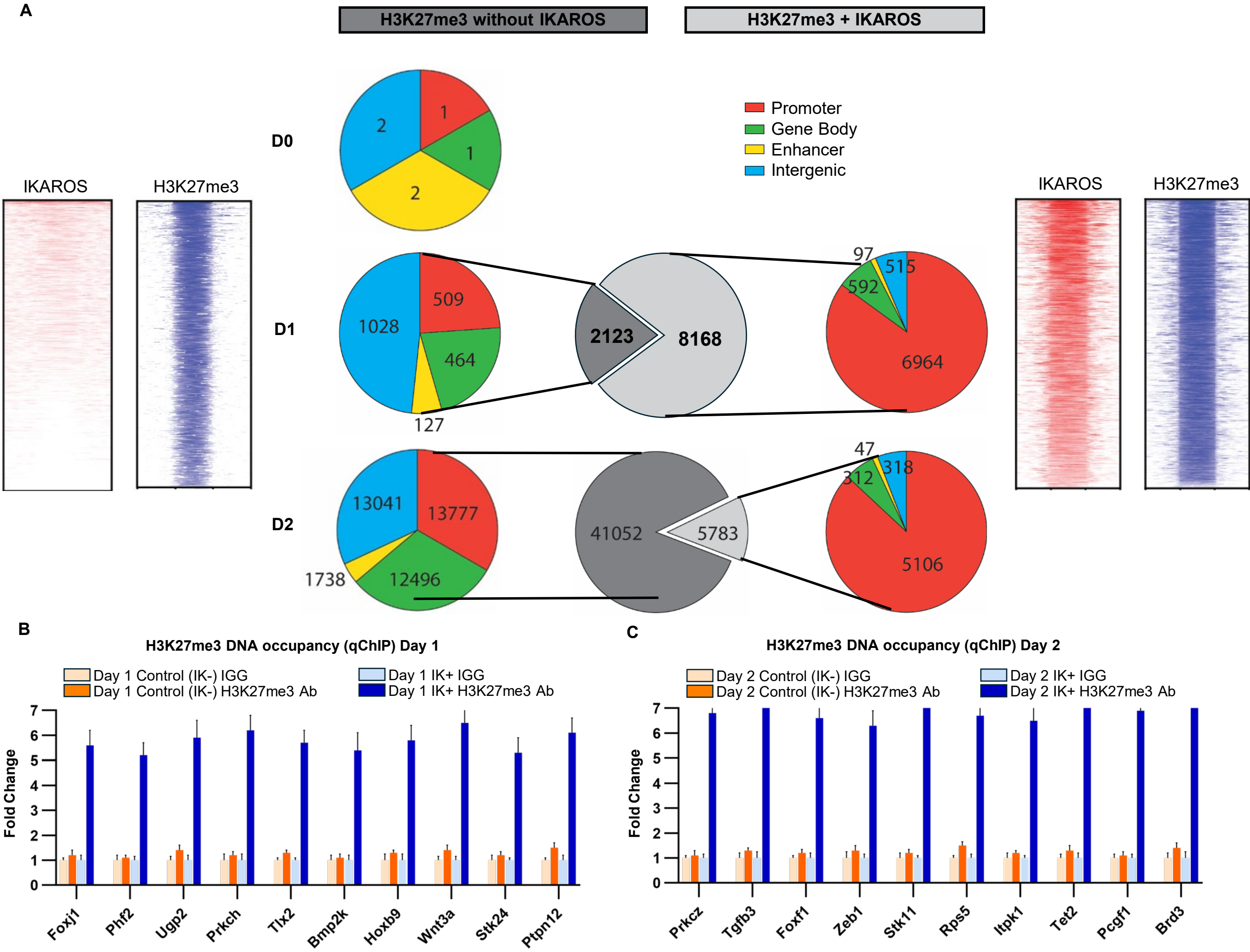

Supplemental Figure 3. Dynamic changes in genome-wide distribution of IKAROS binding-associated facultative heterochromatin (H3K27me3). (A) Dynamic changes in IKAROS binding-associated facultative heterochromatin (right-panel) and facultative heterochromatin formed following *Ikzf1* re-introduction but not associated with Ikaros occupancy in untreated Ikaros-null T-ALL (Day 0) and for 2 Days following *Ikzf1* re-introduction (Day 1 and Day 2). At Day 0 there are very few distinct H3K27me3 peaks. At Day 1 after transduction with *Ikzf1*, the majority (8,168) of the de novo formed facultative heterochromatin (H3K27me3 peaks) are associated with Ikaros occupancy (right panel) as opposed to 2,128 H3K27me3 peaks which are not associated with IKAROS occupancy (left panel). In contrast, at Day 2 after transduction with *Ikzf1*, there is a large increase in the total number of H3K27me3 peaks, with the majority of the new H3K27me3 peaks (41,052) not being associated with IKAROS occupancy (left lower panel), while 5,783 H3K27me3 peaks were associated with IKAROS binding (right lower panel). During both Days after *Ikzf1* transduction, Ikaros binding-associated H3K27me3 are predominantly located at gene promoters, while H3K27me3 peaks not associated with IKAROS binding are relatively evenly distributed among promoters, gene body and intergenic regions, with a significant number of H3K27me3 peaks detected within enhancer regions in Day 2 (lower left panel – yellow). Heat maps for IKAROS binding-associated (right) and IKAROS binding-independent (left) H3K27me3 peaks are shown. **(B)-(C) IKAROS expression is associated with facultative heterochromatin (H3K27me3) at promoters of specific genes**. *Ikzf1* -null T-ALL were transduced with empty MSCV-IRES-GFP retrovirus (control cells - IK-) or with *Ikzf1*-containing MSCV-IRES-GFP retrovirus (IK+ cells). Analysis of the H3K27me3 enrichment at the promoters at specific genes was performed on control cells (orange and red bars) and on *Ikzf1*-null T-ALL following *Ikzf1* re-expression (light blue and dark blue bars). H3K27me3 enrichment was determined using qChIP with anti-H3K27me3 antibodies (red and dark blue bars) and normalized to the IGG background (orange and light blue bars). qChIP analysis of H3K27me3 enrichment was performed on control and IK+ cells at **(B)** 1 day and **(C)** 2 days following Ikaros re-expression. Graphed data are the mean +/– SD of triplicates representative of one of 3 independent experiments.

Figure S4 Dynamic changes in H3K27me3 signature during the first 2 Days following *Ikzf1* re-introduction is consistent with H3K27me3 landscape in physiological mouse thymocyte

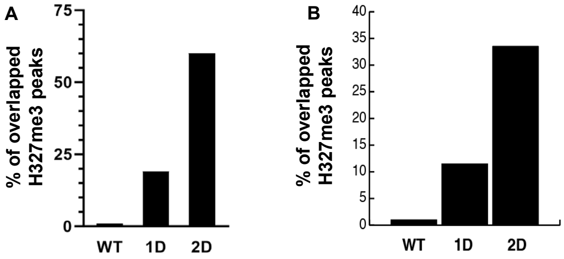

Supplemental Figure 4. Re-introduction of *Ikzf1* into *Ikzf1*-null T-ALL progressively restores normal H3K27me3 landscape. Comparison of global genomic distribution H3K27me3 peaks in *Ikzf1*-null T-ALL 1 day and 2 days following *Ikzf1* re-expression with H3K27me3 landscape in **(A)** mouse thymocytes and **(B)** human thymocytes (published data GSM4083595). Results show that *Ikzf1* re-introduction over 2 Days period significantly restores H3K27me3 landscape, which at the end of 2 Days overlaps with **(A)** 60% of H3K27me3 peaks in normal mouse thymocytes and **(B)** with over 33% of H3K27me3 peaks in normal human thymocytes at DN3 stage of differentiation.

Fig S5. IKAROS is essential for physiological H3K27me3 landscape

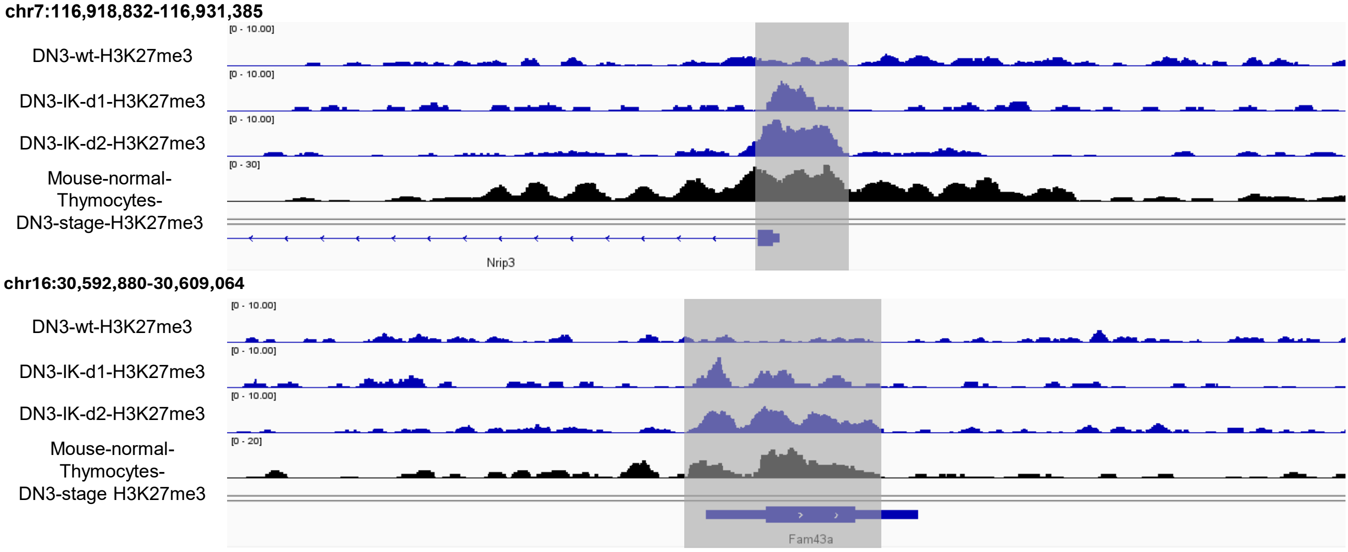

Supplemental Figure 5. Example of the dynamic restoration of normal H3K27me3 landscape in IK-null T-ALL following *Ikzf1* re-introduction. Comparison of published H3K27me3 landscape in normal thymocytes at DN3 stage of differentiation (bottom panels) with IK-null T-ALL and following *Ikzf1* re-introduction in Day 1 and 2 (top 3 panels) - example. Results show that normal H3K27me3 landscape is erased in IK-null T-ALL, but *Ikzf1* re-introduction restores normal (physiological) H3K27me3 landscape.

Figure S6 EZH2-IKAROS complexes are required for H3K27me3 formation

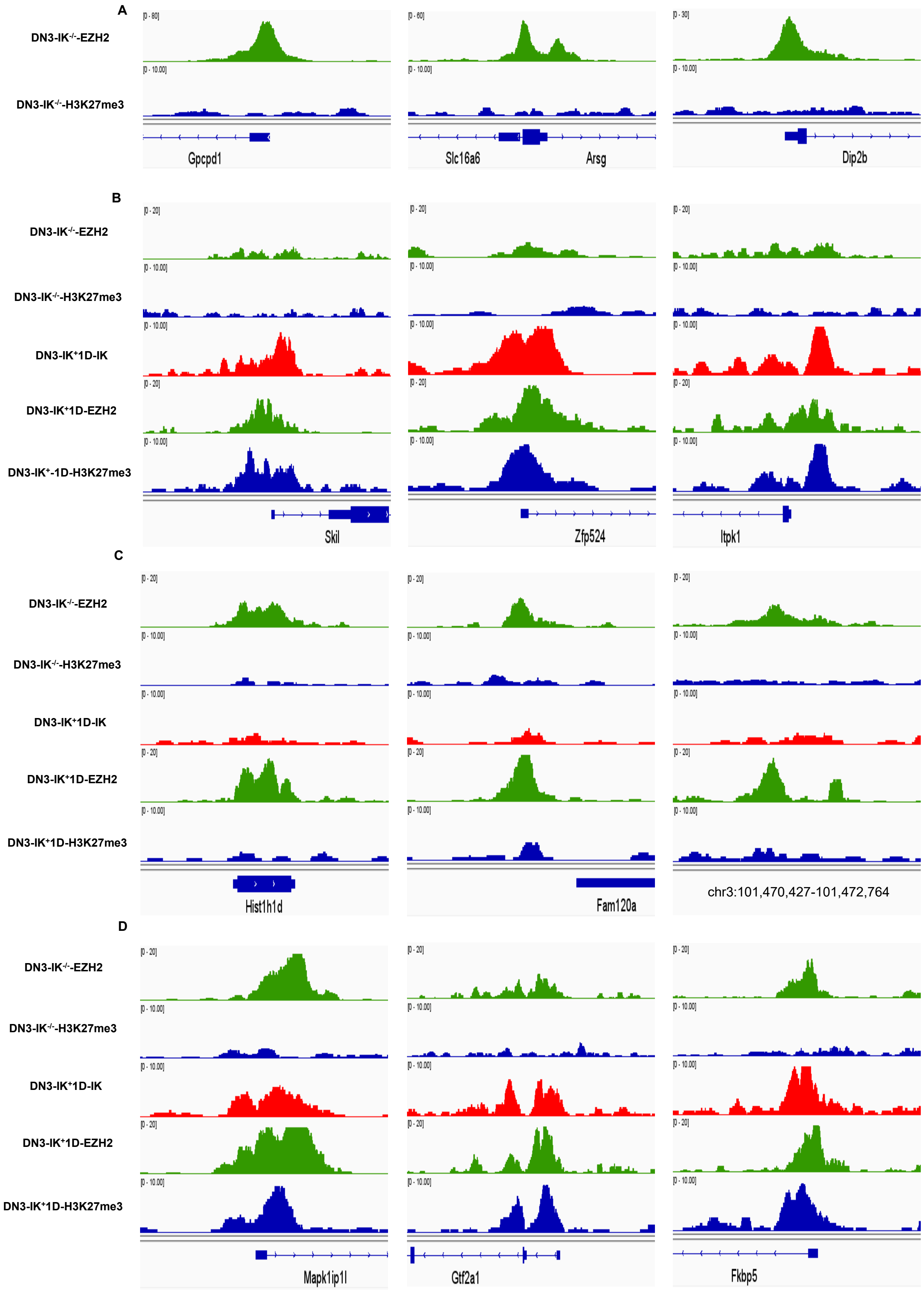

Supplemental Figure 6. (A) Examples of EZH2 DNA binding in *Ikzf1*-null T-ALL without H3K27me3 occupancy; **(B)** Recruitment of EZH2 by IKAROS results in de novo formation of EZH2-IKAROS DNA-binding complexes in IK$^+$Day 1 following *Ikzf1* re-introduction, resulting in formation of H3K27me3 (Day 0 EZH2 not binding, no H3K27me3; IK$^+$Day 1 IKAROS binding, EZH2 binding H3K27me3 formation); **(C)** EZH2 binding to DNA without concomitant IKAROS binding, 1 Day following *Ikzf1* re-introduction is not associated with H3K27me3 (Day 1 EZH2 peak, no Ikaros peak , no H3K27me3 peak). **(D)** Sites occupied solely by EZH2 in Ik-null T-ALL, and not being enriched in H3K27me3, following formation of IKAROS-EZH2 complexes in Day #1, result in H3K27me3 enrichment – EZH2 binding in Day 0; no H3K27me3 Day 0, IKAROS binding in Day 1, EZH2 binding in Day 1 and H3K27me3 peak in Day 1.

Figure S7 Relationship between EZH2 and IKAROS peaks in *Ikzf1*-null T-ALL and 1 Day following *Ikzf1* re-introduction

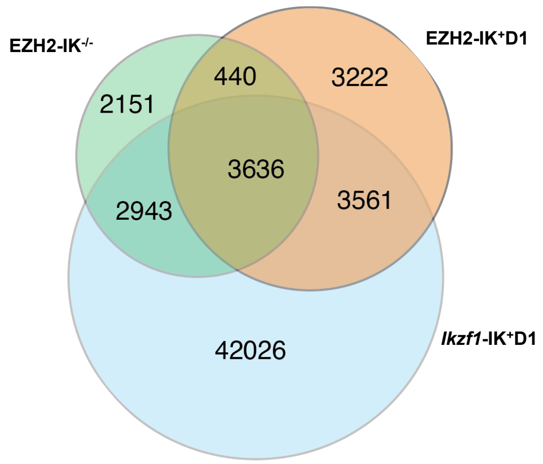

Supplemental Figure 7. Genome distribution of EZH2 ChIP-seq peaks in Ikaros-null T-ALL (Day 0) and 1 Day after *Ikzf1* transduction (D1), as well as IKAROS ChIP-seq peaks 1 Day after *Ikzf1* transduction. Results show that IKAROS binding results in redistribution of EZH2 genome occupancy either by direct recruitment by IKAROS (3,561 peaks), or via IKAROS-independent manner (3,222 peaks). A large number (3,636) EZH2 binding sites, that are occupied in *Ikzf1*-null T-ALL are bound by IKAROS following *Ikzf1* transduction, resulting in formation of EZH2-IKAROS complexes.

Fig S8. Dynamic changes of EZH2 DNA Occupancy

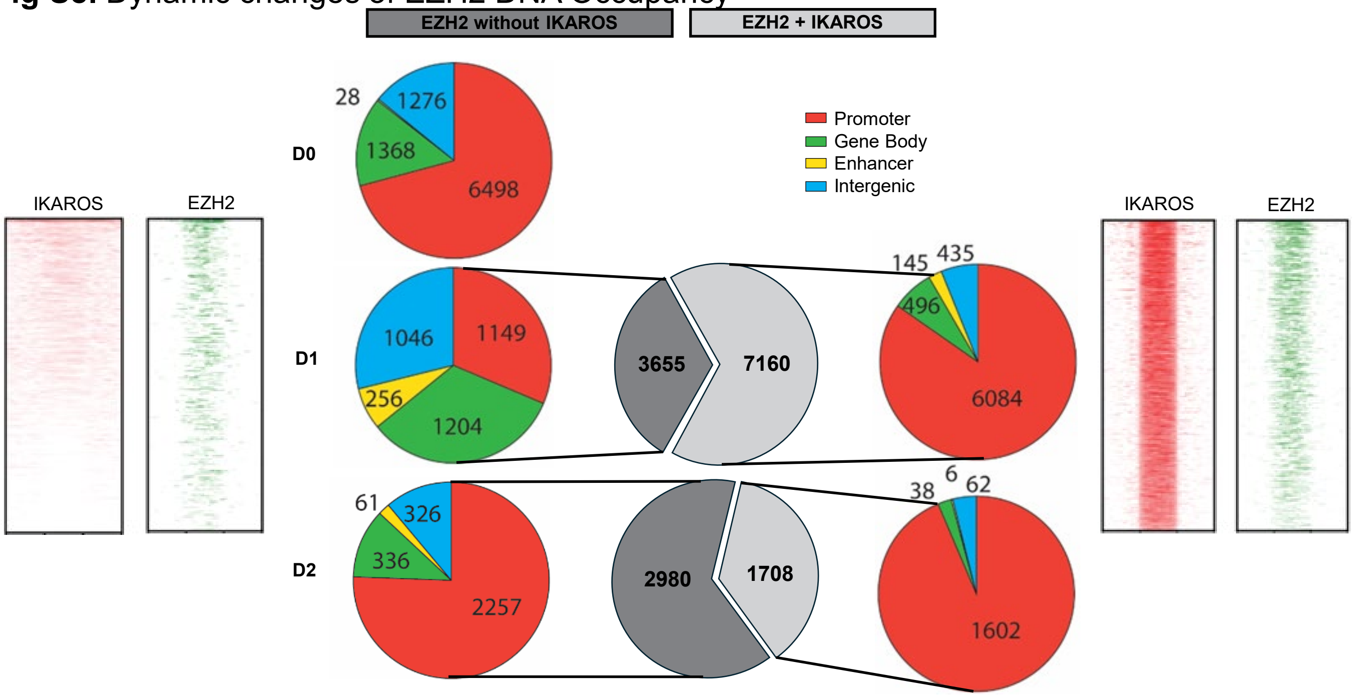

Supplemental Figure 8. Dynamic changes EZH2-only and EZH2-IKAROS occupied sites classified by function of the DNA element. Dynamic changes of EZH2 DNA binding sites in *Ikzf1*-null T-ALL (top panel) and EZH2-IKAROS and facultative heterochromatin formed following *Ikzf1* re-introduction but not associated with IKAROS occupancy in untreated *Ikzf1*-null T-ALL (Day 0) and for 2 s following *Ikzf1* re-introduction (day 1 and day 2). At Day 0 there are very few distinct H3K27me3 peaks. At Day 1 after transduction with *Ikzf1*, the majority (Day8,168) of the de novo formed facultative heterochromatin (H3K27me3 peaks) are associated with IKAROS occupancy (right panel) as opposed to 2,128 H3K27me3 peaks which are not associated with IKAROS occupancy (left panel). In contrast, at Day 2 after transduction with *Ikzf1*, there is a large increase in the total number of H3K27me3 peaks, with the majority of the new H3K27me3 peaks (41,052) not being associated with IKAROS occupancy (left lower panel), while 5,783 H3K27me3 peaks were associated with IKAROS binding (right lower panel). During both Days after *Ikzf1* transduction, IKAROS-binding associated H3K27me3 are predominantly located at gene promoters, while H3K27me3 peaks not associated with IKAROS binding are relatively evenly distributed among promoters, gene body and intergenic regions, with a significant number of H3K27me3 peaks detected within enhancer regions in Day 2 (lower left panel – yellow). Heat maps for IKAROS binding-associated (right) and IKAROS binding-independent (left) H3K27me3 peaks are shown.

Fig S9. Dynamic changes of HDAC1 DNA binding

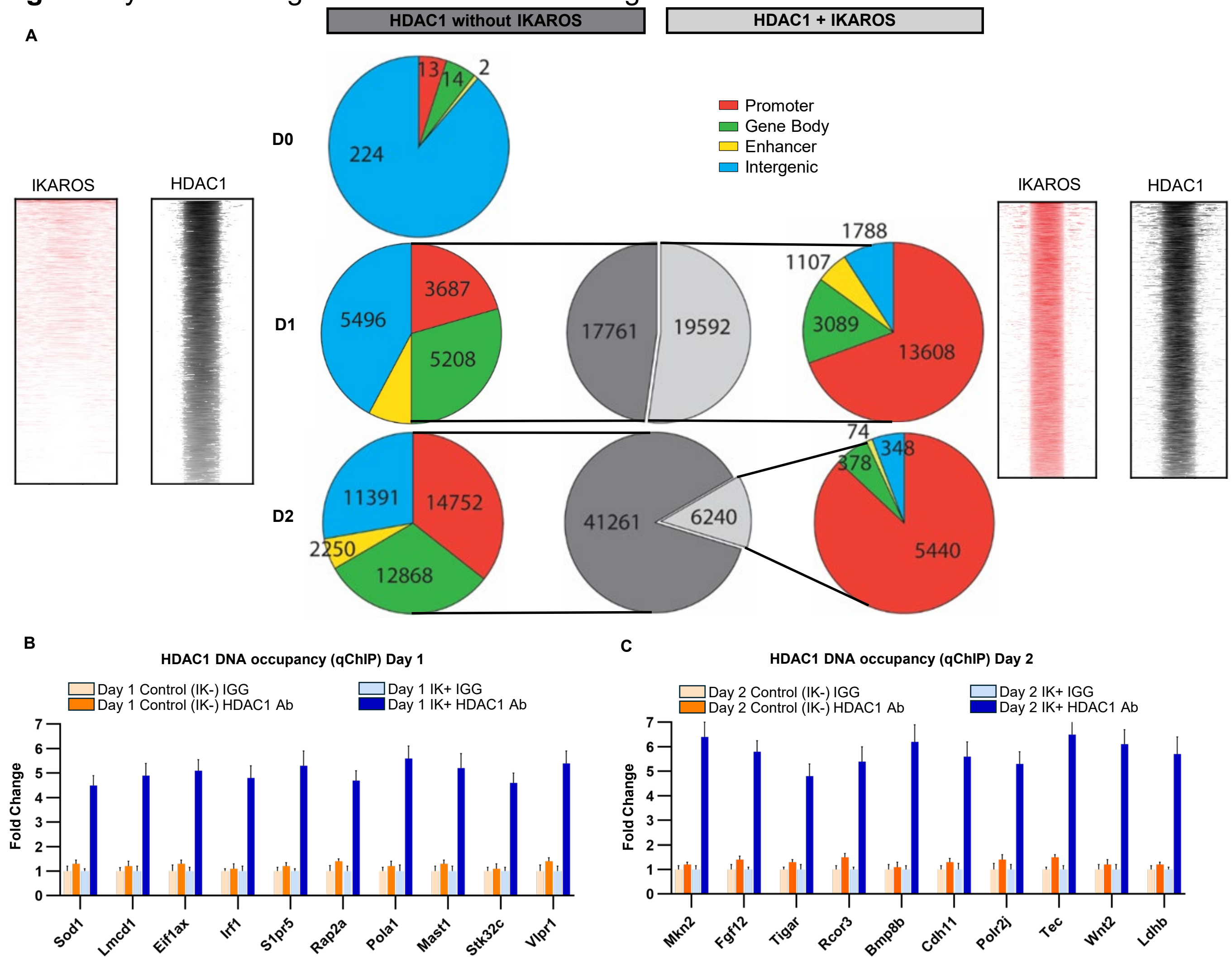

Supplemental Figure 9. Dynamic changes HDAC1-only and HDAC1-IKAROS occupied sites classified by function of the DNA element. Dynamic changes in DNA occupancy of HDAC1 without IKAROS (left) and HDAC1-IKAROS complexes (right) are show in *Ikzf1*-null T-ALL (Day 0) and for 2 Days following *Ikzf1* re-introduction (Day 1 and Day 2). At Day 0 there are very few HDAC1peaks. At Day 1 after transduction with *Ikzf1*, over half (19,592) of the HDAC1 peaks are associated with IKAROS occupancy (right panel) as opposed to 17,761 HDAC1 peaks which are not associated with IKAROS occupancy (left panel). In contrast, at Day 2 after transduction with *Ikzf1*, there is a large increase in the total number of HDAC1 peaks, with the majority of the new HDAC1 peaks (41,261) not being associated with IKAROS occupancy (left lower panel), while 6,240 HDAC1 peaks were associated with IKAROS binding (right lower panel). During both Days after *Ikzf1* transduction, IKAROS-HDAC1 complexes are predominantly located at gene promoters, while HDAC1 peaks not associated with IKAROS binding are relatively evenly distributed among promoters, gene body and intergenic regions, with a significant number of HDAC1 peaks detected within enhancer regions in Day 2 (lower left panel – yellow). Heat maps for IKAROS binding-associated (right) and IKAROS binding-independent (left) HDAC1 peaks are shown. **(B)-(C) IKAROS expression is associated with HDAC1 occupancy at promoters of specific genes**. *Ikzf1*-null T-ALL were transduced with empty MSCV-IRES-GFP retrovirus (control cells - IK-) or with *Ikzf1*-containing MSCV-IRES-GFP retrovirus (IK+ cells). Analysis of the HDAC1 enrichment at the promoters at specific genes was performed on control cells (orange and red bars) and on *Ikzf1*-null T-ALL following *Ikzf1* re-expression (light blue and dark blue bars). HDAC1 enrichment was determined using qChIP with anti-HDAC1 antibodies (red and dark blue bars) and normalized to the IGG background (orange and light blue bars). qChIP analysis of HDAC1 enrichment was performed on control and IK+ cells at **(B)** 1 day and **(C)** 2 days following *Ikzf1* re-expression. Graphed data are the mean +/– SD of triplicates representative of one of 3 independent experiments.

Figure S10

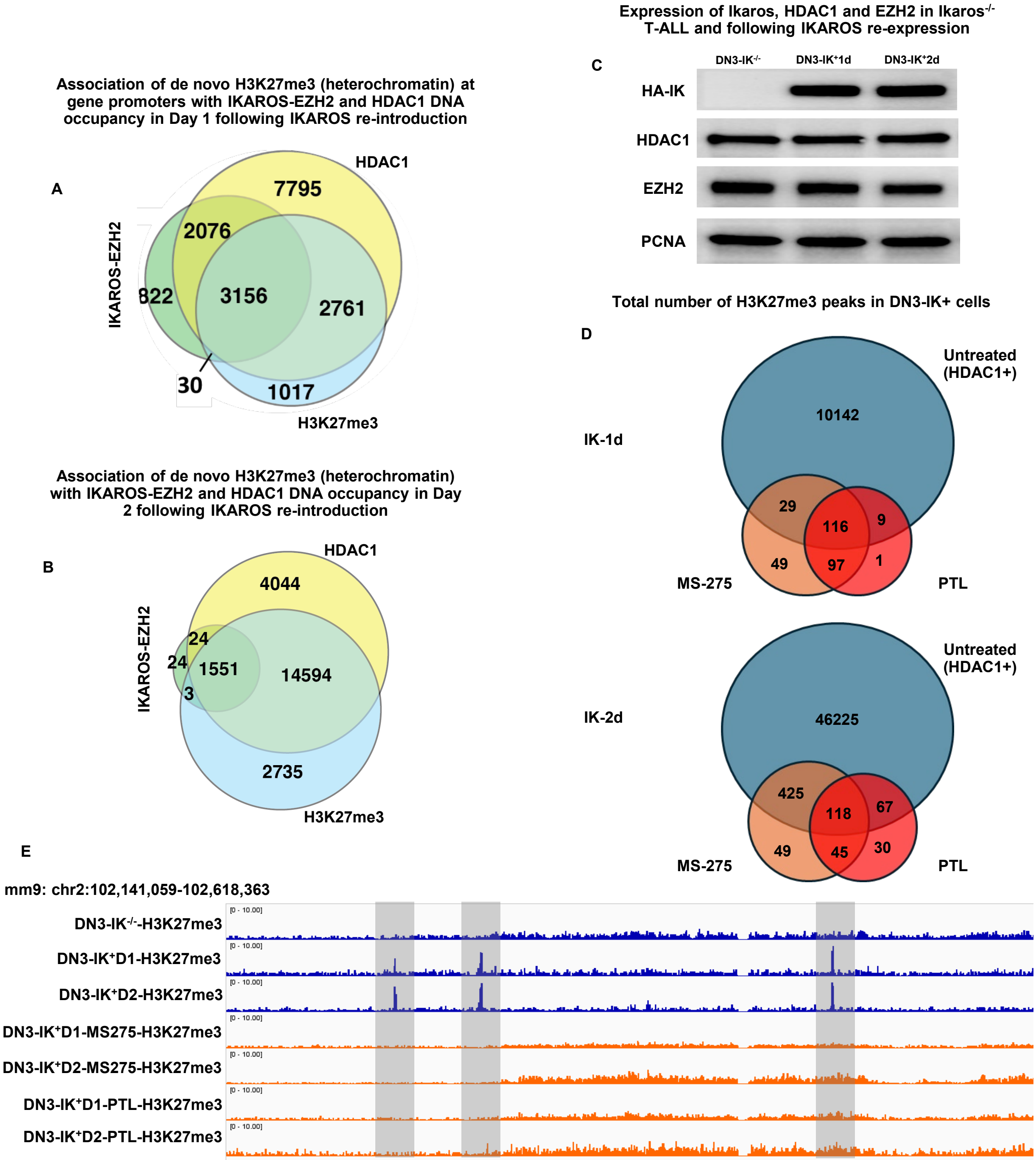

Supplemental Figure 10 Association of Ikaros, EZH2 and HDAC1 with de novo formation of H3K27me3 at promoters. Results demonstrate a predominant role of HDAC1 in de novo formation of H3K27me3. **(A)** In Day 1 following *Ikzf1* expression, formation of H3K27me3 at promoters is similarly associated with IKAROS, EZH2 and HDAC1 co-occupancy (3,156 peaks) and with HDAC1-only occupancy (2,761 peaks). **(B)** In contrast, in Day2 following *Ikzf1* re-introduction, most of the H3K27me3 peaks at promoters (14,594) are associated with HDAC1-only DNA binding. Notably, no H3K27me3 peaks are associated with EZH2-only DNA binding, further supporting the hypothesis that EZH2 H3K27me3 methyltransferase activity requires IKAROS and/or HDAC1 DNA occupancy. **(C)** Western blot analysis shows that expression level of HDAC1 and EZH2 are similar in DN3 IK$^{-/-}$ and following *Ikzf1* re-expression. PCNA expression is used as a loading control. **(D) HDAC1 inhibition severely reduces H3K27me3 DNA enrichment.** The impact of HDAC1 inhibition on H3K27me3 enrichment in Ikzf1-re-expressing T-ALL cells was assessed by using two different HDAC1 inhibitors – MS-275 and (-)-Parthenolide (PTL). The effect of MS-275 and PTL HDAC1 inhibitors was tested on *Ikzf1*-re-expressed T-ALL cells at two different time points - 1 day (top panel) and 2 days (bottom panel) following *Ikzf1* re-expression in T-ALL. The ChIP-seq results show the total number of H3K27me3 peaks (at promoters and other parts of genome) in *Ikzf1* re-expressing T-ALL (blue) and following 4 hours inhibition with 1 μmol of MS-275 or PTL (orange/red). Results show that inhibition of HDAC1 by each of two inhibitors at both 1 day and 2 days following *Ikzf1* re-expression in T-ALL severely reduces number of H3K27me3 peaks. **(E) Example of the impact of HDAC1 inhibition on H3K27me3 DNA enrichment in IK-null T-ALL following *Ikzf1* re-introduction.** ChIP-seq of H3K27me3 peaks in *Ikzf1*-null T-ALL with *Ikzf1* re-expressing T-ALL 1 and 2 days following *Ikzf1* re-expression, and the *Ikzf1* re-expressing T-ALL cells treated with MS-275 or PTL. Results showed that *Ikzf1* re-expression is associated with H3K27me3 peaks at 1 day following *Ikzf1* re-expression (DN3-IK$^+$D1-H3K27me3) and 2 days following *Ikzf1* re-expression (DN3-IK$^+$D2-H3K27me3). Treatment of those cells with 1 μM of MS-275 for 4 hours at 1 day following *Ikzf1 re-expression* (DN3-IK$^+$D1-MS275-H3K27me3) and 2 days following *Ikzf1 re-expression* (DN3-IK$^+$D2-MS275-H3K27me3) or with 1 μM of PTL for 4 hours at 1 day following *Ikzf1 re-expression* (DN3-IK$^+$D2-PTL-H3K27me3 and DN3-IK$^+$D2-PTL-H3K27me3) is associated with the loss of H3K27me3 peaks. Areas surrounding H3K27me3 peaks are highlighted grey.

Figure S11

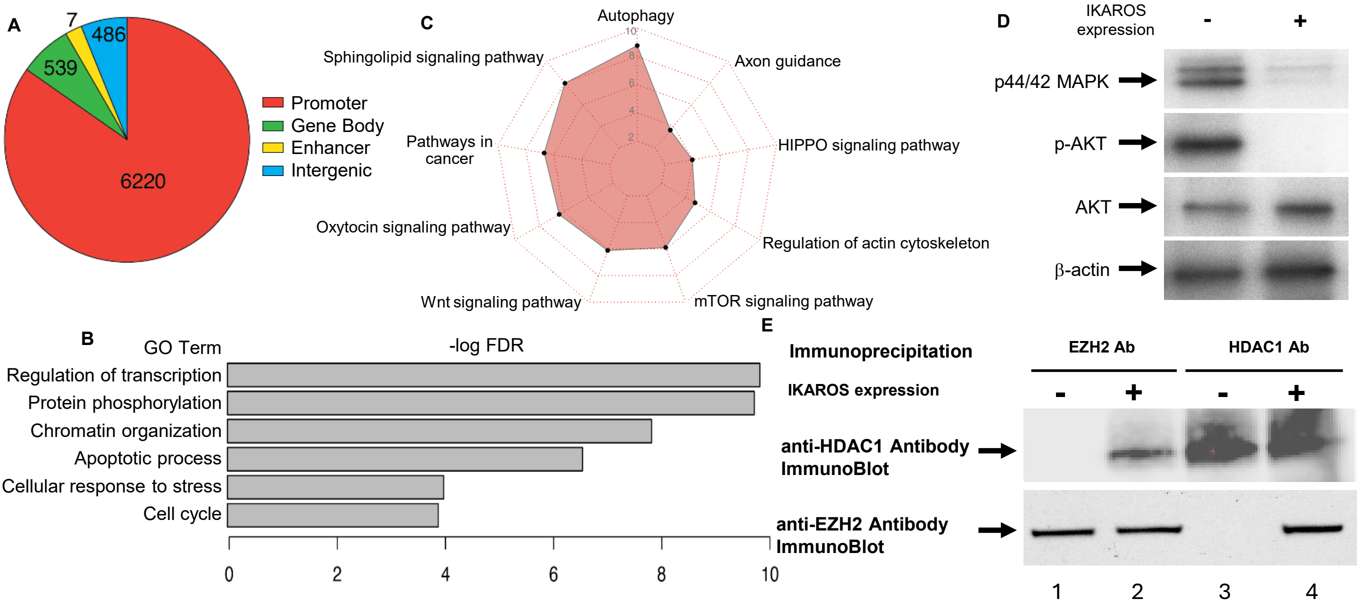

Supplemental Figure 11. EZH2 target genes that are regulated by IKAROS/HDAC1 induced epigenetic switch. (A) Distribution of de novo induced facultative heterochromatin (H3K27me3) at EZH2-occupied sites via IKAROS and HDAC1-induced epigenetic switch following *Ikzf1* re-introduction to *Ikzf1*-null T-ALL. **(B-C)** Gene ontology and pathway enrichment analysis of EZH2 target genes that are regulated by IKAROS and HDAC1-induced epigenetic switch. Many pathways that are regulated by IKAROS/HDAC1-induced epigenetic switch are known for their oncogenic activity. Results suggest that *Ikzf1*-null T-ALL results in functional inactivation of EZH2, lack of H3K27me3 occupancy, and activation of the above oncogenic pathways. IKAROS/HDAC1-induced switch to H3K27me3 occupancy results in repression of oncogenic pathways and tumor suppression. **(D)** Analysis of the effect of *Ikzf1* re-expression on cancer signaling pathways. *Ikzf1*-null T-ALL transduced with empty MSCV-GFP retrovirus (control cells-left) and T-ALL cells transduced with MSCV-GFP retrovirus-containing *Ikzf1* (right) were analyzed for Western blot with p44/42 MAPK, and pAKT antibodies to evaluate activity of MAPK and AKT pathways. Results showed that IKAROS expression negatively regulates both MAPK and AKT pathway. **(E) EZH2-HDAC1 interaction is dependent on IKAROS expression.** Co-immunoprecipitation analysis of EZH2 and HDAC1 interaction. To determine whether the IKAROS expression is essential for interaction between HDAC1 and EZH2, co-immunoprecipitation experiments for HDAC1 and EZH2 were performed in *Ikzf1*-null T-ALL (control cells, lanes 1 and 3) and in T-ALL following *Ikzf1*-re-expression (lanes 2 and 4). Cells were lysed in NETN buffer without EDTA and subjected to IKAROS or HDAC1 immunoprecipitations with anti-IKAROS or anti-HDAC1 Antibodies. The pellet fractions were loaded in a 9% SDS-PAGE with the following order: Lanes 1-2: Immunoprecipitation with anti-EZH2 antibodies of control cels (Lane 1) and T-ALL with *Ikzf1* re-expression (Lane 2). Lanes 3-4: : Immunoprecipitation with anti-HDAC1 antibodies of control cels (Lane 3) and T-ALL with *Ikzf1* re-expression (Lane 4). The gel was blotted and analyzed by immunoblotting with anti-EZH2 or anti-HDAC1 antibodies indicated to the left of each panel to show the binding of EZH2 and HDAC1 proteins. Results demonstrate that HDAC1 interacts directly with EZH2 in *Ikzf1*-expressing T-ALL, but not in *Ikzf1*-null T-ALL. These data suggest that IKAROS expression is critical for HDAC1-EZH2 interaction

Figure S12 Genes regulated by Active Enhancers occupied by IKAROS without HDAC1 – 2,391 target genes

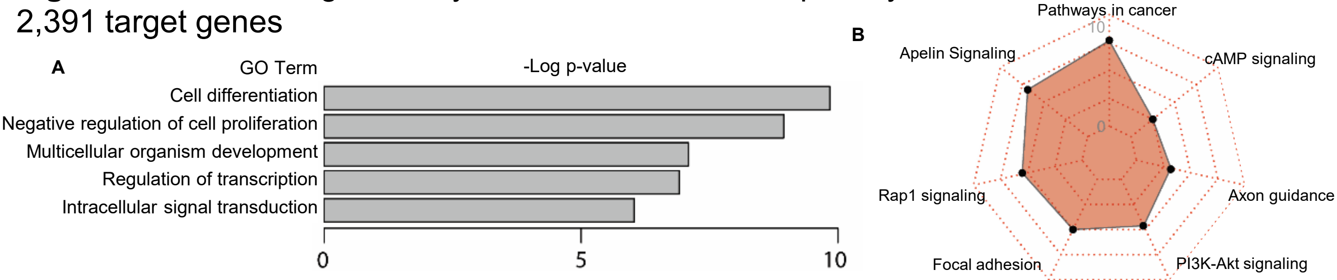

Supplemental Figure 12. (A) Gene ontology and (B) pathway enrichment analysis of the genes that are regulated by active enhancers occupied by IKAROS but not by HDAC1

Figure S13 Genes regulated by Active Enhancers occupied by HDAC1, but not by IKAROS – 1,425 genes

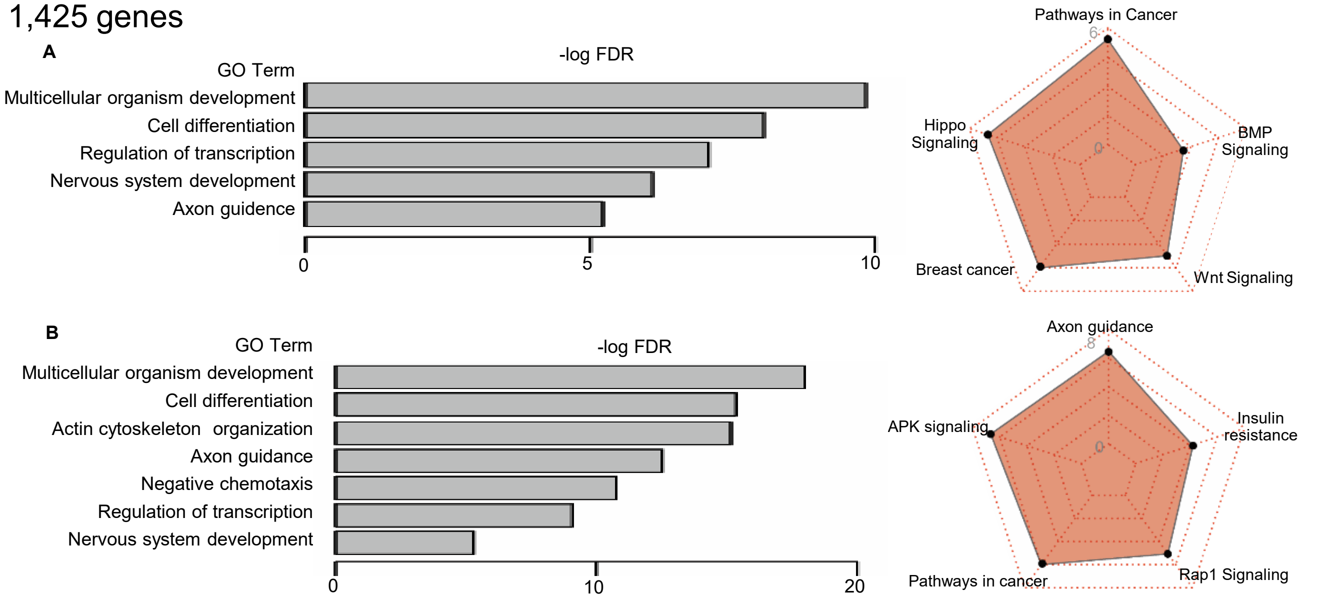

Supplemental Figure 13. Gene ontology (left) and pathway enrichment analysis (right) of the genes that are regulated by active enhancers occupied by both HDAC1 but not IKAROS in **(A)** Day 1 and **(B)** Day 2 following *Ikzf1* re-introduction

Figure S14 Genes regulated by Active Enhancers occupied by both IKAROS and HDAC1 – 1,667 target genes

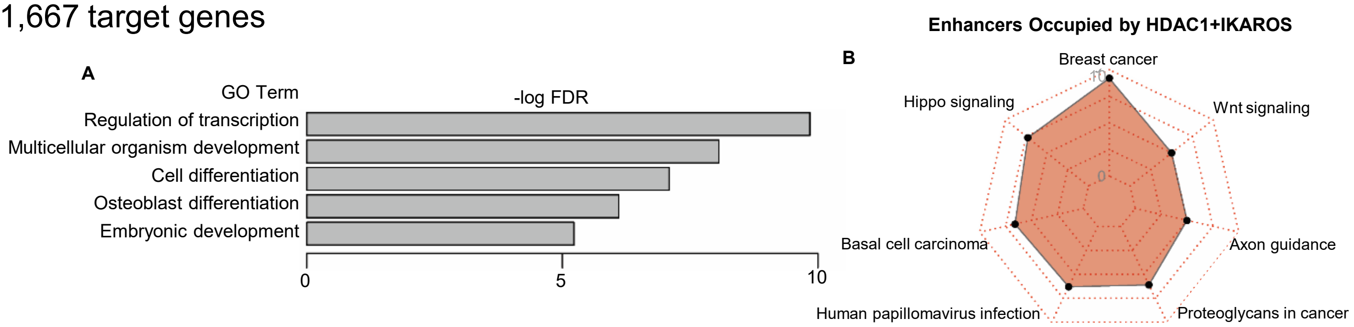

Supplemental Figure 14 (A) Gene ontology and **(B)** pathway enrichment analysis of the genes that are regulated by active enhancers occupied by both IKAROS and HDAC1

Figure S15 Genes regulated by Active Enhancers in *Ikzf1*-null T-ALL, which becomes silenced by IKAROS and/or HDAC1 following Ikaros re-introduction – 276 target genes

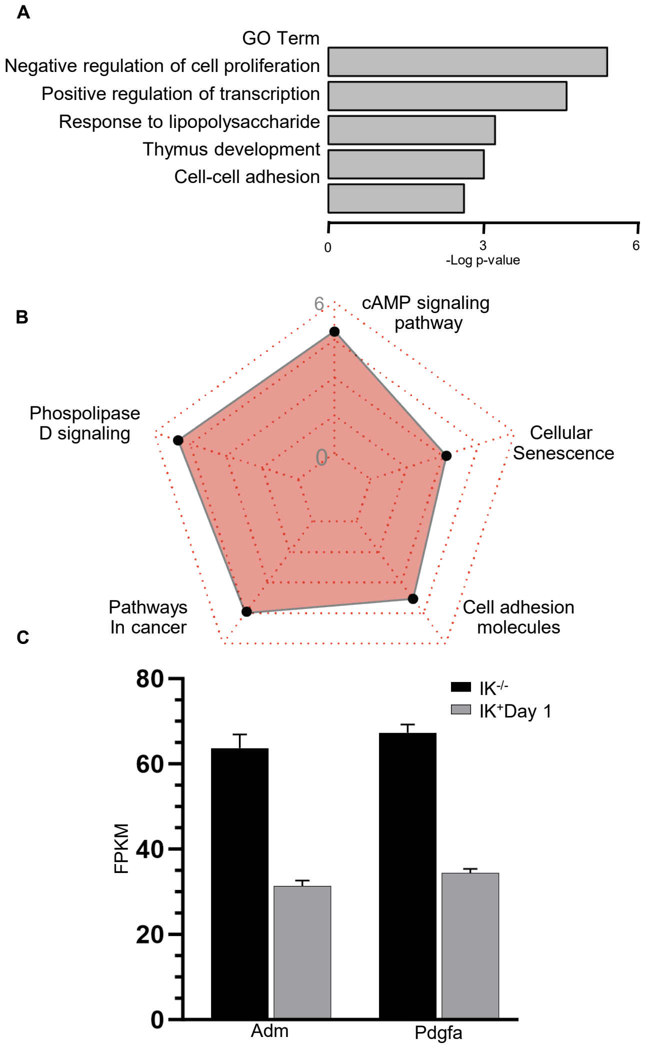

Supplemental Figure 15 (A) Gene ontology **(B)** pathway enrichment analysis of genes regulated by Ikaros$^{-/-}$ T-ALL, which became silenced by IKAROS and/or HDAC1 following *Ikzf1* re-expression. **(C)** examples of gene repression due to enhancer silencing by IKAROS and HDAC1 following *Ikzf1* re-expression.

Fig S16. Associated genes with K27me3 LOCKs

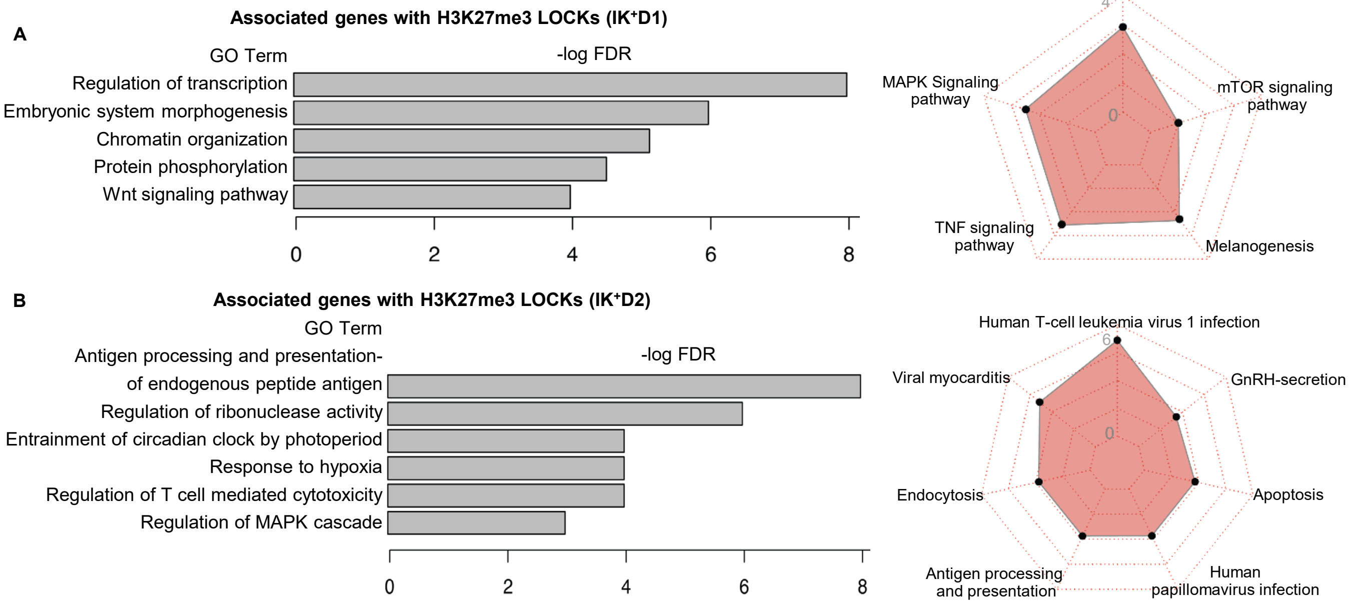

Supplemental Figure 16. Gene ontology (left) and pathway enrichment analysis (right) of the genes that are found within the H3K27me3 LOCKs in **(A)** Day 1 and **(B)** Day 2 following *Ikzf1* re-expression

Fig S17 Human MOLT-4 wt vs. MOLT-4 IK-KO

A

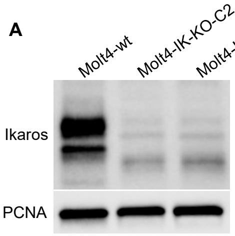

C

**MAPK**					**Wnt**			**TGF-B**
JUN	TAB2	MKNK2	HSPA8	MKNK1	JUN	NFATC3	CACYBP	ROCK1
ELK1	TGFBR1	RAP1A	MAP3K14	HSPA1B	PPP3R1	FBXW11	TCF7	RPS6KB1
JUND	MAX	TAOK2	PRKACA	PPM1B	SENP2	RBX1	GSK3B	GDF5
STMN1	DUSP10	PPP3R1	CDC42	MAPK7	ROCK1	CAMK2D	PPP2R1A	PPP2R1A
MAP3K3	RASGRP2	ATF2	SRF	DUSP14	CTNNBIP1	PRKACA	RUVBL1	RBX1
AKT2	MAP2K1	RPS6KA5	RAF1	RRAS	AXIN1	DAAM1	PPP2R5E	TGFBR1
MAPK14	RPS6KA2	DAXX	PAK2	TAOK3	CSNK1A1	CHD8	PPP2R5C	SP1

B

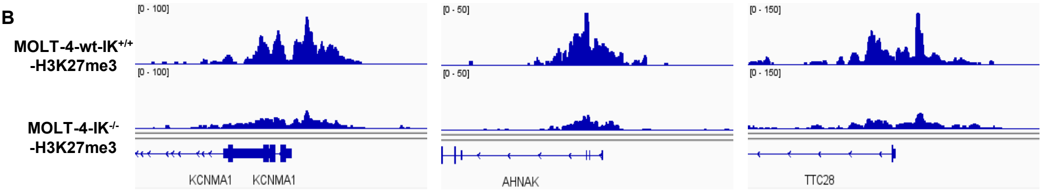

Supplemental Figure 17. (A) IKAROS expression in two different clones of MOLT4-I*KZF1*-null cells. Western blot with anti-IKAROS antibodies was performed in wildtype MOLT4 cells and on two different *IKZF1*-knockout MOLT4 clones. **(B) Example of the loss of H3K27me3 in human T-ALL associated with absence of IKAROS.** Comparison of H3K27me3 CUT&Tag landscape in human T-ALL MOLT-4 cells, which express wild type *IKZF1* (top panel) with H3K27me3 distribution obtained by CUT&Tag on MOLT-4 cells with CRISPR-induced *IKZF1* knockout. Results show that *IKZF1* knockout results in severely reduced H3K27me3 landscape in human T-ALL. (C) The list of the genes involved in MAPK, Wnt and TGF-beta pathway, that have altered expression in MOLT4 *IKZF1*-null cells, compared to the MOLT4 *IKZF1*-wildtype cells.

Fig S18 IKAROS and HDAC1 regulate activity of enhancers in MOLT-4

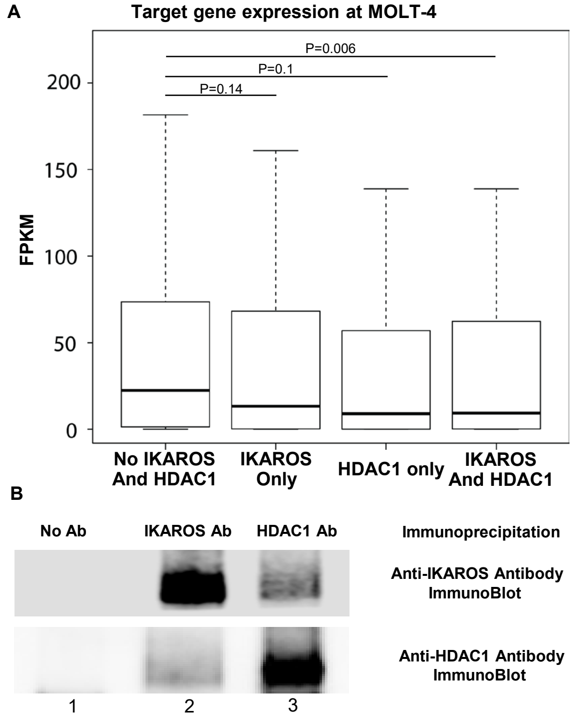

Supplemental Figure 18. IKAROS and HDAC1 occupancy represses active enhancers in human T-ALL. (A) Boxplot shows expression of genes regulated by the active enhancers in human T-ALL not bound by IKAROS and/or HDAC1 (left) and occupied by IKAROS and/or HDAC1. Boxplot: center line represents median, boxes show first and third quartiles, whiskers extend to the most extreme data points that are no more than 1.5-fold of the interquartile range from the box. **(B) IKAROS directly interacts with HDAC1.** Co-immunoprecipitation analysis of IKAROS and HDAC1 interaction. MOLT-4 cells were lysed in NETN buffer without EDTA and subjected to IKAROS or HDAC1 immunoprecipitations with anti-IKAROS or anti-HDAC1 Antibodies. The pellet fractions were loaded in a 9% SDS-PAGE with the following order: Lane 1: Cell extract with no antibodies as negative control. Lane 2: cell extracts immunoprecipitated with IKAROS antibody. Lane 3: cell extracts immunoprecipitated with HDAC1 antibody. The gel was blotted and analyzed by immunoblotting with anti-IKAROS or anti-HDAC1 antibodies indicated to the right of each panel to show the binding of IKAROS and HDAC1 proteins.

Fig S19 IKAROS regulates formation and expansion of H3K27me3 LOCKs in human T-ALL

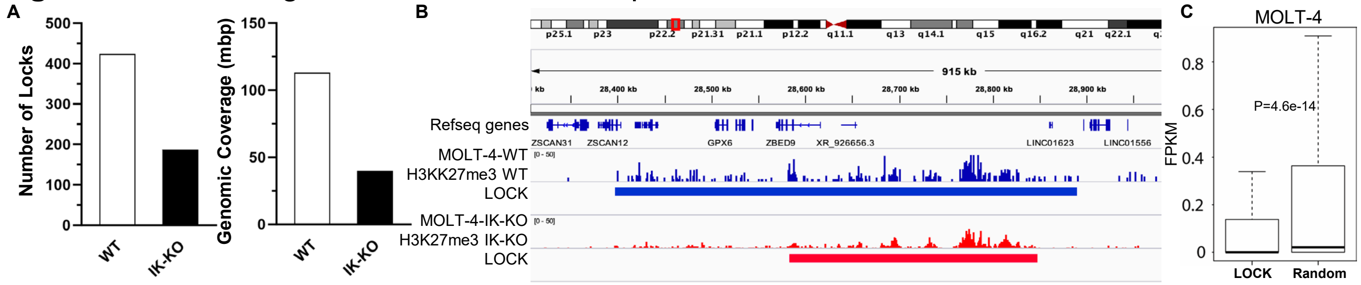

Supplemental Figure 19. IKAROS regulates formation and expansion of H3K27me3 LOCKs in human T-ALL. (A) Number (left) and genomic coverage (right) of H3K27me3 LOCKs in human T-ALL with wildtype *IKZF1* (IK-wt) and *IKZF1* knockout (IK-KO) **(B)** Example of the genomic expansion of the H3K27me3 LOCKs in human T-ALL with wildtype *IKZF1* (IK-wt) vs. human T-ALL with *IKZF1* knockout (IK-KO). **(C) Negative regulation of gene by LOCKs.** Expression of genes located within LOCK compared to the expression of the genes not regulated by LOCKS.

Fig S20 IKAROS regulates large set of genes in human T-ALL via formation of H3K27me3 LOCKs

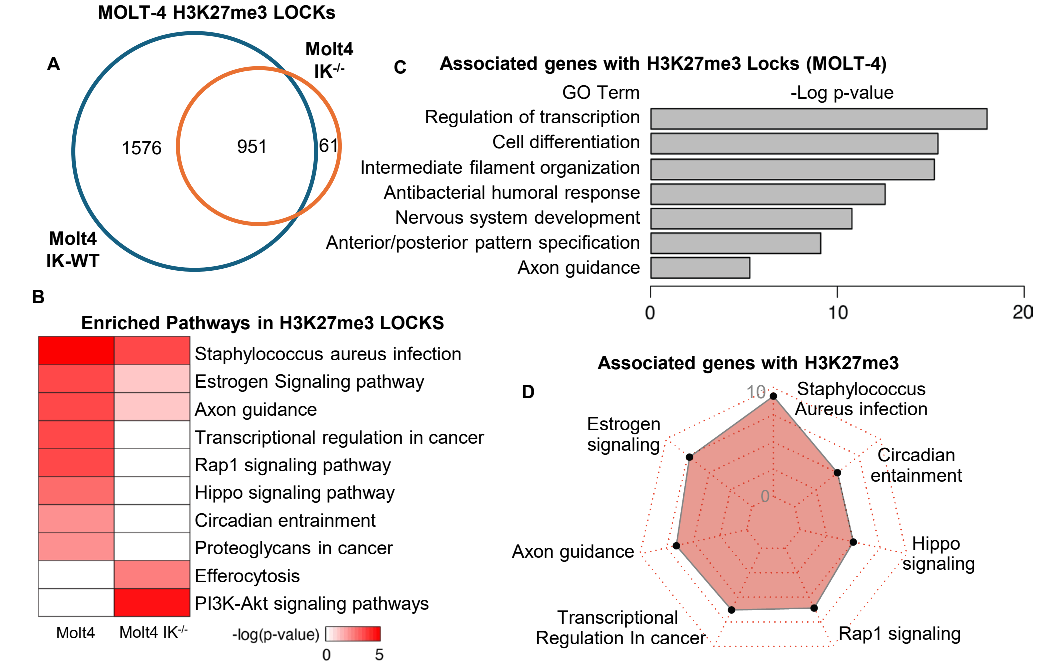

Supplemental Figure 20 IKAROS regulates large set of genes in human T-ALL via formation of H3K27me3 LOCKs **(A)** Number of genes regulated by the H3K27me3 LOCKs in human T-ALL with wildtype *IKZF1* (IK-wt) and *IKZF1* knockout (IK-KO). **(B)** Gene set enrichment analysis on the genes found within the H3K27me3 LOCKs in human T-ALL with wildtype *IKZF1* (IK-wt) and *IKZF1* knockout (IK-KO). **(C)** Gene ontology and **(D)** Pathway analysis of the genes found within H3K27me3 LOCKs in human T-ALL with wildtype *IKZF1*.

Fig S21 BGRD vs random in WT

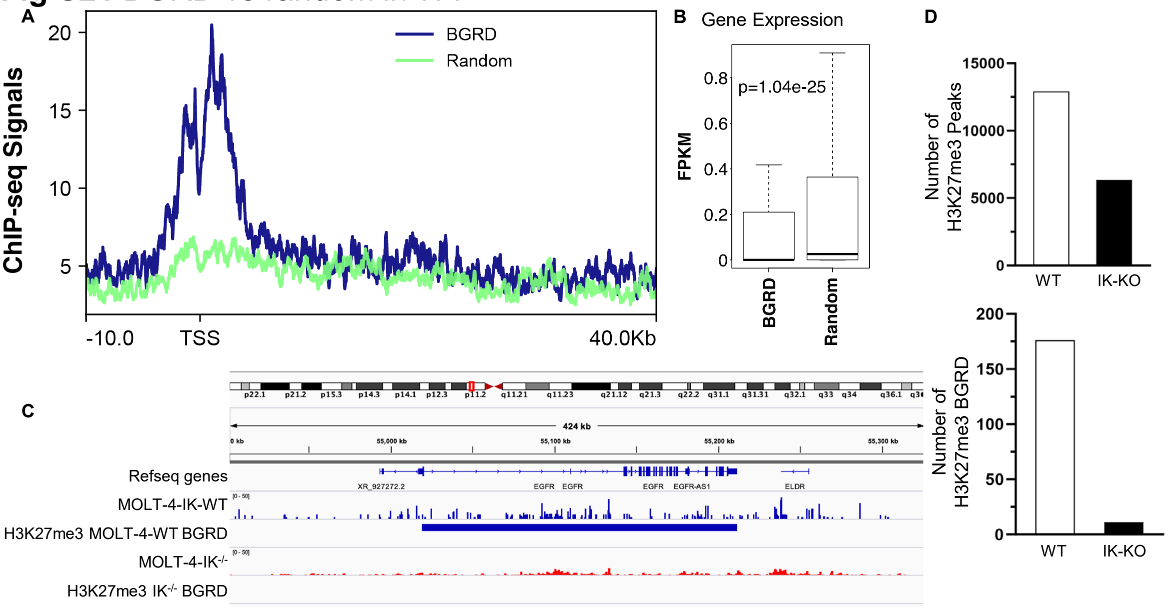

Supplemental Figure 21 IKAROS regulate formation of H3K27me3 BGRDs in human T-ALL. (A) Average ChIP-seq signal value for H3K27me3 plotted around TSS of the genes within BGRD region vs. the random genes **(B)** Boxplot shows the expression value of the genes (by RNA-seq) located within BGRD vs. random genes. Center line is median, while boxes show first and third quartiles, with whiskers extending to the most extreme data points that are no more than 1.5-fold of the interquartile range from the box. **(C)** Example of BGRD which include EGFR oncogene in human T-ALL with wildtype *IKZF1* (IK-WT) vs. human T-ALL with *IKZF1* knockout (IK-KO). **(D)** List of the oncogenes and tumor suppressor genes associated with H3K27me3 BGRDs in human T-ALL with wildtype *IKZF1* (IK-WT) vs. human T-ALL with *IKZF1* knockout (IK-KO).

Fig S22 Mouse vs human Thymocytes and T-ALL

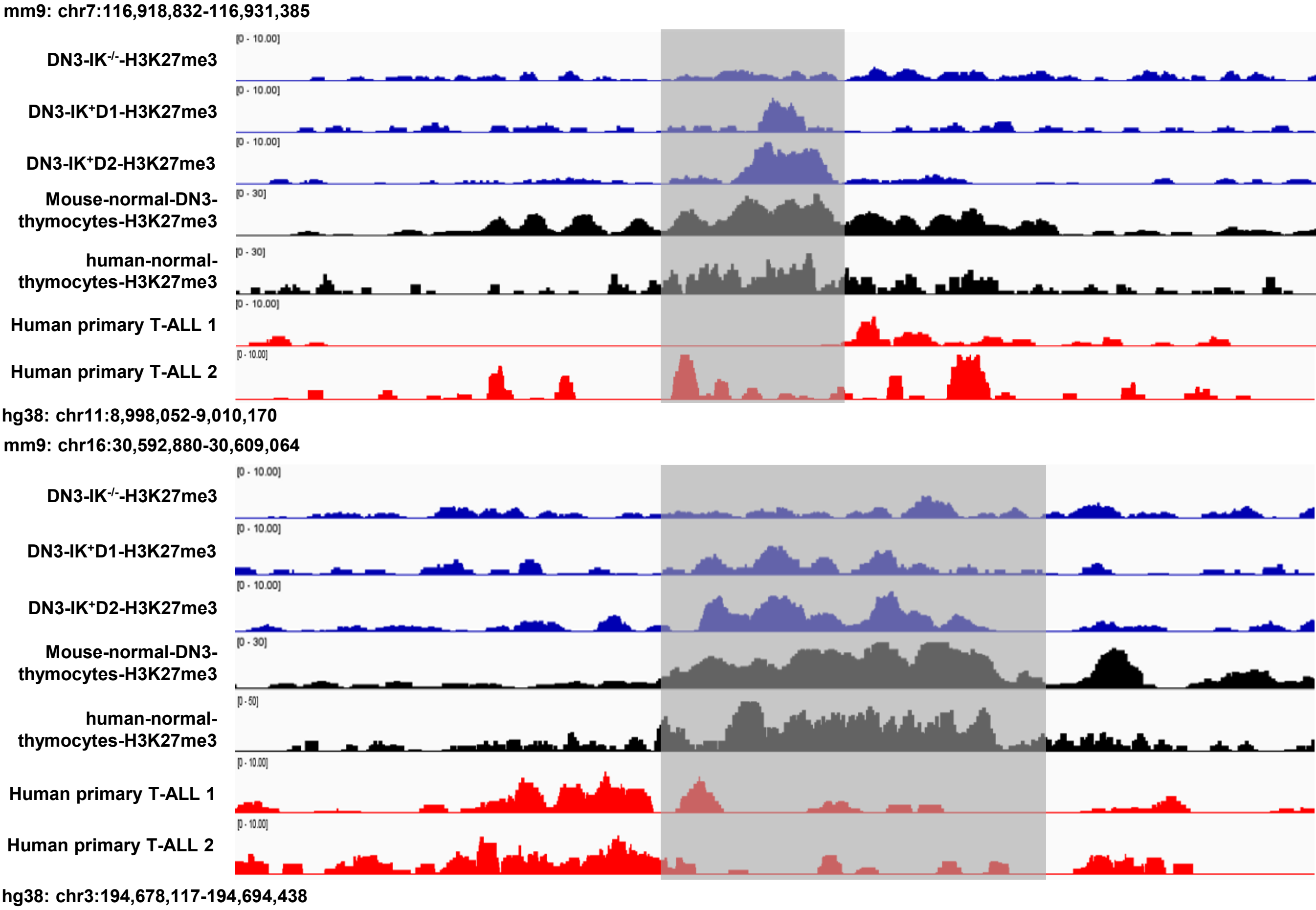

Supplemental Figure 22. Example of conservation of H3K27me3 heterochromatin in mouse *Ikzf1*-null and two human T-ALL (T-ALL 1-2), normal mouse and human thymocytes, and the role of IKAROS in re-establishing normal H3K27me3 landscape. Results show loss of H3K27me3 signature in mouse and human T-ALL (top and bottom panel). Re-introduction of *Ikzf1* into mouse Ikaros-null T-ALL results in partial re-establishment of the normal H3K27me3 landscape that is observed in mouse and human thymocytes. ChIP-seq for H3K27me3 was done by our team (top 6 samples), while the H3K27me3 ChIP-seq of the bottom sample was published previously (from GEO accession GSM1431912).

Methods

Supplemental Methods

Cell culture

Molt4 Ikaros CRISPR knockout and control cells were purchased form Synthego, Redwood City, CA. The sequence of sgRNA for knockout Ikaros gene is AUCUGGAGUAUCGCUUACAG. Cells were cultured with RPMI 160 with 10% FBS. Both PCR and western blot were performed to confirm the knockout of Ikaros. Human primary T-ALL PDX cells W8 were obtained from Loma Linda University (Loma Linda, CA) in compliance with Institutional Review Board regulations.

The JE131 cell line (referred to here as DN3) is an early T-ALL cell line derived from Ikaros knock-out mice that spontaneously develop T-ALL and is not commercially available. Cells are tested monthly for mycoplasma infection and have been found to be negative for infection. Both wild-type DN3 cells and retrovirally-transduced cells were maintained in RPMI 1640 medium with 10% heat-inactivated FBS at 37℃ with 5% CO2.

Western blot

10^6 cells were collected and lysed by Pierce IP Lysis Buffer (Pierce, 87787) on ice for 10 minutes. Cell debris were removed by centrifugation at 13, 000g at 4℃ for 10 minutes. Supernatants were transferred to new tubes for Western blot which was performed according to standard procedure. Antibodies which were used include anti-HA tag (Abcam, ab9110) for HA-tagged Ikaros, anti-HDAC1 (Abcam, ab7028), anti-EZH2 (Active Motif, 39901) and anti-PCNA (Santa Cruz, sc-7907).

ChIP-seq

Antibodies that were used including anti-HA tag (Abcam, ab9110) for HA-tagged IKAROS, anti-HDAC1 (Abcam, ab7028) and anti-histone modification antibodies: H3K4me3 (Abcam, ab8580), anti-H3K4me1(Abcam, ab8895), anti-H3K27ac (Abcam, ab4729) and anti-H3K27me3 (Millipore, 07-449). ChIP-seq libraries were created using ChIP-seq DNA sample prep kit (Illumina), size-selected and the 200-400bp fraction was extracted and purified. Libraries were sequenced at the High Throughput Genomics Center of University of Washington, Seattle and at Genome Sciences and bioinformatics core of Penn State University, Hershey, College of Medicine.

CUT&Tag

CUT&Tag for W8 and MOLT-4 cells was performed according to EpiCypher CUTANA Direct-to-PCR CUT&Tag Protocol. Briefly, approximately 100k cells for each sample were collected. Nuclei were extracted by NE buffer and were then immobilized to Concanavalin A conjugated paramagnetic beads (EpiCypher, 21-1401). Then the immobilized nuclei were incubated with primary then secondary antibodies. pAG-Tn5 (EpiCypher, 15-1017) were added to recognize antibodies which were the same for ChIP-seq, cleave target-DNA complex, and ligate sequencing adapters. Then DNA fragments were amplified by PCR, and purified by Agencourt AMPure XP beads (Beckman Coulter Genomics, #A63881). Sample libraries were sequenced on NovaSeq 6000 PE150 at Novogene, Sacramento, CA.

Bioinformatics Analysis

ChIP-seq Analysis

Both histone and transcription factor ChIP-seq data were analyzed using ENCODE3 pipeline. Briefly, Bowtie2 (ref. 1) (version 2.2.6) with default parameters to map fastq data to the mm9 reference genome. Samtools (ref. 2) (version 1.2) with MAPQ > 30 as a cutoff and PICARD (version 2.0.1) were used for further filtering. MACS2 (ref. 3) (version 2.1.1) was used to call peaks, followed by Benjamini-Hochberg procedure with q values of 0.05 as a cutoff for significance. For Transcription factor ChIP-seq, IDRR (ref. 4) (version 2.0.4) was further used to control the reproducibility between replicates with a cutoff value of 0.05. Finally, Narrowpeak files and p value bigwig signal files were used for downstream analysis.

Histone ChIP-seq peaks were used to define DNA elements. Promoters were defined as either H3K4me3 peak regions or 1.5kb upstream and downstream of TSS of genes defined in the annotation GTF file with GENCODE release version vM1. Poised Enhancers were defined as H3K4me1 peak regions that do not overlap with Promoter regions. Active Enhancers were defined as regions that have both H3K4me1 and H3K27Ac peaks. Gene Body regions were defined as annotation with the GENCODE release version vM1 but do not overlap with either Promoters or Enhancers. Ikaros regulated DNA elements were defined by overlapping Ikaros peaks with each of above-mentioned DNA elements. The remaining Ikaros binding regions were defined as Gene Desert regions.

De novo enhancers were defined as regions that gained H3K4me1 signals with Ikaros treatment as compared to wild type regardless of Ikaros binding or H3K27Ac signals. De novo activated enhancers were defined as a subset of de novo enhancers that also gained H3K27Ac signals. Ikaros regulated de novo enhancers are the subset of de novo enhancers that gained Ikaros binding when Ikaros-treated cells were compared with untreated cells.

To compare the functions of DNA elements across species, we used liftOver to convert the coordinates of the identified DNA elements from mouse genome (mm9) to human genome (hg38).

Super-enhancers, Enhancers, LOCKs, and BGRDs

Super-enhancer identification was defined using HOMER (version 4.8). Firstly, peaks for H3K27Ac were found as described above. Then, peaks within 10 000 bp were combined together into larger regions. The H3K27Ac signals of these merged peak regions were then determined by the total input normalized number of reads as well as highest score and the total number of enhancer regions. Finally, intensity was potted against the rank of these enhancers, super-enhancers are identified as regions past the point where the tangent of slope is greater than 1, the rest would be typical enhancers.

Large organized chromatin lysine (K) domains (LOCKs) and broad genic repression domains (BGRDs) were identified following the original publications. To identify LOCKs, we first used MACS2 to identify H3K27me3 peaks in DN3 cells. And then we used CREAM algorithm to identify the peak clusters as LOCK (ref. 5). To identify BGRDs, after peak calling for H3K27me3 peaks, we retrieved the peaks that overlapped with each gene. We then plotted the maximal height of H3K27me3 peaks against the total width of H3K27me3 peaks for each gene. We finally used an H3K27me3 width of 120kb as the cutoff to define BGRDs (ref.6).

ATAC-seq analysis

ATAC-seq data were analyzed using the ENCODE3 ATAC-seq pipeline. Briefly, raw fastq data were firstly trimmed using CUTADAPT (ref. 7) (version 1.9.1) and then mapped to the hg19 reference genome with Bowtie2 (ref. 1) (version 2.2.6) using default parameters (-k -X2000 --local --mm --threads -x). Post-alignment filtering was achieved using Samtools (ref. 2) (version 1.2) with MAPQ > 30 as a cutoff and removing duplicate reads using PICARD (version 2.0.1). MACS2 (ref. 3) (version 2.1.1) was used for calling peaks with q values of 0.05 as a cutoff. Finally, narrowpeak files and p value bigwig signal files were used for downstream analysis. Ikaros-regulated de novo open chromatin regions were defined by a gained ATAC-seq signal after treatment, as compared with untreated. Functional categories of these de novo open regions were determined by overlapping them with each category of DNA elements defined above.

RNA-seq analysis

RNA-seq read data were aligned to mm9 reference genome using STAR (version 2.7.7) with –r 100 –no-coverage-search. FeatureCounts (ref. 8) (version 1.4.6) was then used to get read counts for each transcript. Symbol conversion was conducted through biomaRt (ref. 9) package (version 2.30.0). Data from three technical replicates were merged for subsequent analysis. Genes with less than 100 reads were filtered. The top-ranked differential regulated genes were determined by fold change. Welch's two-sample t-test was used to determine the statistical significance between genes from two conditions. Volcano plots were generated in R using –log p-value plot against log fold change value. Significantly differentially expressed genes ($p < 0.05$) are color-coded: genes up-regulated by >2 fold are in orange, genes down-regulated by >2 fold are in purple, genes up or down regulated by <2 fold but with $p < 0.05$ are in red.

Enhancer-target gene prediction

We used a recent developed algorithm Integrated Method for Predicting Enhancer Targets (IM-PET) to predict enhancer targets (ref.10). IM-PET predicts enhancer-promoter by integrating four features using a Random Forest classifier. Features are derived from transcriptomic, epigenomic and genome sequence data, including enhancer and promoter activity correlation, TF and promoter activity correlation, enhancer and promoter sequence co-evolution and enhancer-to-promoter distance. We showed that IM-PET achieved significant improvement over other state-of-the-art methods. Further, based on our validation experiment using 3C-qPCR we showed that IM-PET has a comparable accuracy to that of the experimental 5C technology. Here, the input data for IM-PET included the genomic positions of predicted enhancers, RNA-Seq data and H3K4me1, H3K4me3, and H3K27Ac ChIP-Seq data for DN3 cells and Molt4 cells. Enhancer targets were predicted using a false discovery rate cutoff of 0.01.

Heatmaps, boxplots, volcano plots and radar plots

Enrichment of transcription and Histone ChIP-seq signals in specific regions were analyzed and visualized using deeptools (ref. 11) (version 2.3.5) python package. Briefly, input normalized and p value filtered bigwig files generated from the ENCODE pipeline were used to compute a matrix for genomic regions of interest using computeMatrix function. In the matrix, each row is a genomic region and each column contains normalized chip-seq reads for each transcription factor/histone at a specific time point. The matrix was used as input for plotHeatmap function to generate heatmaps. Track view of examples for enhancers and super enhancers were generated using the UCSC genome browser with bigwig file as input. Volcano plots were generated in R using –log p-

value against log fold change value. Significantly differential expressed genes are color coded as described in figure legends. Boxplots and radar plots were also generated in R.

Motif enrichment analysis

Motif enrichment analysis was conducted using MEME Suite (ref. 12) (version 5.0.1) http://meme-suite.org/ with database set to HOMOCOMO human (v11 core). Briefly, genomic sequence in fasta format for ChIP-seq peak regions were extracted using bedtools (version 2.25.0) from mm9 reference genome. MEME-ChIP function was then used to test the enrichment of known transcription factor-binding motifs in the database between target DNA sequences and randomly generated sequences. The length of each motif was set to a range from 3 to 10 bp. The minimum searching threshold was set as an E value of 0.05.

GO term and pathway enrichment analysis

GO term and pathway enrichment analysis was achieved using The Database for Annotation, Visualization and Integrated Discovery (DAVID) (ref. 13) (version 6.8) https://david.ncifcrf.gov/. FDR cutoff 0.05 was used to define significant enrichment. For cis-regulatory regions, both Genomic Regions Enrichment of Annotations Tool (GREAT) (ref. 14) (version 3.0.0) http://great.stanford.edu/public/html/ and assignment with the closest TSS followed by a similar procedure as for gene approaches was used. Redar plot was conducted using –log10 of adjusted p values.

GSEA analysis

Gene Set Enrichment Analysis (GSEA) software was downloaded from the Broad Institute website http://software.broadinstitute.org/gsea/index.jsp. Molecular Signatures Database (v6.1 MSigDB) was used to test enrichment for target genes with specific function. Briefly, the target set of genes were firstly ordered based on mean differential expression values of the two classes divided by the sum of the standard deviations. Then, an enrichment measure based on normalized Kolmogorov-Smirnov statistic was calculated for each gene set. The genes from a set with previously defined known function were computed by a running sum across all target genes. The ES enrichment score is defined as the maximum observed positive deviation of the running sum. ES is measured for every functional gene set considered. In this manuscript, after identifying Ikaros regulated genes, we tested it (a single gene set) for association with functions associated with T cells using GSEA.

Data access

RNA-seq, ChIP-seq and ATAC-seq libraries were sequenced by Genome Sciences and bioinformatics core at Penn State University, Hershey, College of Medicine. All sequencing data are available as fastq-format files from the GEO archive under accession numbers GSE261180 and GSE261181

HDAC inhibitors:

Two different HDAC1 inhibitors were used - MS275 (Entinostat, Ambeed Cat# A122285), and (-)-Parthenolide (PTL, Ambeed Cat# A105353) – a specific HDAC1 inhibitor, which specifically depletes HDAC1 protein without affecting other class I/II HDACs. The effect of each HDAC1 inhibitor was tested on *Ikzf1*-re-expressed T-ALL cells at two different time points (1 day and 2 days) each inhibitor was used at 1 μM concentration, for 4 hours.

.

Validation of ChIP-Seq by qChIP

qChIP experiments were performed, and fold change of Ikaros binding relative to control IgG was calculated. Primers for qChIP were as following:

References

1. Langmead B, Salzberg SL. Fast gapped-read alignment with Bowtie 2. Nat Methods. 2012;9(4):357-9.

2. Li H.* HB, Wysoker A., Fennell T., Ruan J., Homer N., Marth G., Abecasis G., Durbin R. and 1000 Genome Project Data Processing Subgroup The Sequence alignment/map (SAM) format and SAMtools. Bioinformatics. 2009(25):2078-9.

3. al Ze. Model-based Analysis of ChIP-Seq (MACS). . Genome Biol. 2008;9(9):137.

4. al Le. ChIP-seq guidelines and practices of the ENCODE and modENCODE consortia. Genome Res. 2012(22):1813-31.

5. Madani Tonekaboni SA, Haibe-Kains B, Lupien M. Large organized chromatin lysine domains help distinguish primitive from differentiated cell populations. Nat Commun. 2021;12(1):499.

6. Zhao D, Zhang L, Zhang M, Xia B, Lv J, Gao X, et al. Broad genic repression domains signify enhanced silencing of oncogenes. Nat Commun. 2020;11(1):5560.

7. Martin. M. Cutadapt Removes Adapter Sequences From High-Throughput Sequencing Reads.

8. Liao Y, Smyth GK, Shi W. featureCounts: an efficient general purpose program for assigning sequence reads to genomic features. Bioinformatics. 2014;30(7):923-30.

9. Durinck S SP, Birney E, Huber W Mapping identifiers for the integration of genomic datasets with the R/Bioconductor package biomaRt. . Nature Protocols. 2009;4(4):1184-91.

10. He B, Chen C, Teng L, Tan K. Global view of enhancer-promoter interactome in human cells. Proc Natl Acad Sci U S A. 2014;111(21):E2191-9.

11. Ramirez F, Ryan DP, Gruning B, Bhardwaj V, Kilpert F, Richter AS, et al. deepTools2: a next generation web server for deep-sequencing data analysis. Nucleic Acids Res. 2016;44(W1):W160-5.

12. Timothy L. Bailey MB, Fabian A. Buske, Martin Frith, Charles E. Grant, Luca Clementi, Jingyuan Ren, Wilfred W. Li, William S. Noble. MEME SUITE: tools for motif discovery and searching. Nucleic Acids Research. 2009;37:W202-W8.

13. Huang DW SB, Lempicki RA. Systematic and integrative analysis of large gene lists using DAVID Bioinformatics Resources. Nature Protocols. 2009;4(1):44-57.

14. Cory Y McLean DB, Michael Hiller, Shoa L Clarke, Bruce T Schaar, Craig B Lowe, Aaron M Wenger, and Gill Bejerano. GREAT improves functional interpretation of cis-regulatory regions. Nat Biotechnol. 2010;28(5):495-501.
